# Characterization of Potato Virus Y Isolates and Assessment of Nanopore Sequencing to Detect and Genotype Potato Viruses

**DOI:** 10.3390/v12040478

**Published:** 2020-04-23

**Authors:** Michele Della Bartola, Stephen Byrne, Ewen Mullins

**Affiliations:** Crop Science Department, Teagasc, Oak Park, R93XE12 Carlow, Ireland; michele.dellabartola@teagasc.ie (M.D.B.); stephen.byrne@teagasc.ie (S.B.)

**Keywords:** potato viruses, PVY recombinants, genotype, diversity, sequencing, Nanopore

## Abstract

Potato virus Y (PVY) is the most economically important virus infecting cultivated potato (*Solanum tuberosum* L.). Accurate diagnosis is crucial to regulate the trade of tubers and for the sanitary selection of plant material for propagation. However, high genetic diversity of PVY represents a challenge for the detection and classification of isolates. Here, the diversity of Irish PVY isolates from a germplasm collection and commercial sites was investigated using conventional molecular and serological techniques. Recombinant PVY isolates were prevalent, with PVY^NTNa^ being the predominant genotype. In addition, we evaluated Nanopore sequencing to detect and reconstruct the whole genome sequence of four viruses (PVY, PVX, PVS, PLRV) and five PVY genotypes in a subset of eight potato plants. De novo assembly of Nanopore sequencing reads produced single contigs covering greater than 90% of the viral genome and sharing greater than 99.5% identity to the consensus sequences obtained with Illumina sequencing. Interestingly, single near full genome contigs were obtained for different isolates of PVY co-infecting the same plant. Mapping reads to available reference viral genomes enabled us to generate near complete genome sequences sharing greater than 99.90% identity to the Illumina-derived consensus. This is the first report describing the use of Oxford Nanopore’s MinION to detect and genotype potato viruses. We reconstructed the genome of PVY and other RNA viruses; indicating the technologies potential for virus detection in potato production systems, and for the study of genetic diversity of highly heterogeneous viruses such as PVY.

## 1. Introduction

Potato (*Solanum tuberosum* L.) is an essential food crop cultivated in every continent of the world and a fundamental source of nutrients for many populations in developing countries [1]. In general, potato is susceptible to a high number of pathogens and pests and more than fifty viruses and viroids have been described [2]. Potato is vegetatively propagated, supporting the transmission of viruses over successive generations and making it highly susceptible to damaging viral diseases.

Potato virus Y (PVY) is ranked fifth in the world’s top ten most important plant viruses [3] and considered the most economically harmful virus to cultivated potatoes [4]. It is also responsible for severe diseases in other widely cultivated crops such as tobacco, tomato and pepper [5]. Other than tuber-borne transmission, PVY also has numerous vector aphid species able to transmit the virus in a non-persistent fashion [2,6].

PVY is the type member of the genus *Potyvirus* in the family *Potyviridae* and possesses a single-stranded, positive sense RNA genome of approximately 9.7 kb that encodes a large polyprotein. An additional protein, P3N-PIPO is produced from an overlapping open reading frame [7]. The RNA-dependent RNA polymerase of most RNA viruses lack proofreading activity [8], resulting in a high error-rate during virus replication, which supports the rapid generation of genetic variability and novel variants [9]. In addition, Potyviruses are also well known for their propensity to evolve through recombination [10], with different genotypes of the virus exchanging segments of their genome. PVY exists in nature as a complex of strains and variants that have been characterized and named based on their geographical origin and biological, serological and molecular properties [11,12]. Hundreds of isolates have been sequenced, allowing a clearer understanding of the phylogenetic clades of PVY and the genetic structures of recombinant genotypes [13,14,15,16,17]. However, as more PVY genomes are being sequenced and made available through public databases, the classification and naming of PVY isolates has been continuously developing [12,15,18].

Furthermore, intra-strain genetic variability and ‘rare’ or previously unclassified recombinant genetic structures have been described [14,16], which adds to the complexity. Currently, in addition to five non-recombinant genotypes (PVY^O^, ^-Eu-N^, ^-Na-N^, ^-O5^, ^-C^), thirty-six unique recombinants structures between two or more parental lineages have been reported [14,16,19,20,21,22,23,24,25,26,27,28,29,30,31]. Certain PVY recombinants are often associated with the induction of potato tuber necrotic ringspot disease (PTNRD) in susceptible potato cultivars [13,32,33], which can make tubers unmarketable. Among them, PVY^NTN^ genotypes have been reported from many areas of the world [24,30,31,33,34,35,36,37,38,39] and represent a relevant concern for potato production. PVY^O^ used to be the predominant strain infecting potatoes, but recently the recombinant genotypes, once a minor part of the PVY complex, became prevalent in many regions of the world [19,33,34,36,37,39,40,41,42,43,44,45,46,47,48]. This rapid shift in PVY populations could have been unintentionally driven—at least accelerated—by anthropogenic factors, such as seed certification and breeding programs. [33,40,49].

Such impressive genetic diversity confers PVY the ability to survive and prosper in various conditions and hosts [17,50,51] and also challenges the accurate detection of the virus. This impedes correct identification and classification of PVY isolates; thereby impacting the interpretation of biological data to assist in developing Integrated Pest Management (IPM) strategies. In spite of the fact that the diversity of PVY has been investigated in many countries all over the world, these data are still scarce for PVY isolates circulating in Ireland. While previous studies have reported the overall predominance of the PVY^N^ serotype [52] and the presence of recombinant genotypes of the virus [53], finer molecular characterization of the isolates is lacking, and it is still unclear how prevalent the recombinant genotypes are.

Biological indexing by inoculation on several indicator hosts has historically been the main method to discriminate PVY strains and is still essential in defining isolate pathogenicity and the biological properties of an isolate on its hosts [11,24,54]. ELISA (enzyme-linked immunosorbent assay) tests using commercially available polyclonal and monoclonal antibodies are largely used for PVY detection and strain differentiation. However, even though serological assays can distinguish different serotypes of the virus, they are unable to identify recombinant isolates [24]. Several molecular methods relying on RT-PCR and quantitative RT-PCR targeting previously described recombination sites and strain-specific sequences have also been developed and are extensively used for the detection and differentiation of the main PVY genotypes [55,56,57,58,59,60,61]. Even as more and more previously unknown or unclassified genetic structures are being discovered, molecular methods like multiplex RT-PCR can potentially mistype isolates, especially for rare and recently discovered genotypes [16]. Whole genome determination by sanger sequencing of overlapping PCR fragments [14,16,17,26,34,36] or, recently, by next generation sequencing (NGS) [12,43,62] and recombination analysis are recommended to confirm the genetic structure of PVY isolates [16,54].

Since its first use for detection and sequencing [63,64,65] of plant viruses, NGS is becoming widely adopted in plant virology research and diagnostic laboratories. In 2015 Oxford Nanopore Technologies (ONT) released the MinION, a portable single-molecule sequencer and a number of studies have reported on its application to sequence plant viruses [66,67,68,69,70]. While base accuracy of reads generated by ONT sequencing are still relatively low [71], consensus sequences obtained by de novo assembly or by map to reference based approaches have been found to be comparable to Illumina sequencing [67]. Given its economic relevance and its remarkable genetic variability with a plethora of recombinant genotypes, PVY represents a good benchmark to test the performance of ONT sequencing for the detection, genome reconstruction and genotyping of PVY isolates infecting potatoes.

The aim of this study was to investigate PVY diversity in Irish potato production systems through conventional serological and molecular assays, and to assess our ability to reconstruct genomes of PVY and other RNA viruses using short (Illumina) and long read (Oxford Nanopore) sequencing technologies. Recombinant genotypes of PVY were found to be predominant in the samples that we analyzed, and we report the first detection of the PVY^Na-N^ genotype in Ireland. Short and long read sequencing technologies were used to successfully reconstruct the near full genomes of PVY and other RNA viruses. This is the first report describing the use of Oxford Nanopore long read sequencing for detection and molecular characterization of potato viruses. Its ability to detect and efficiently reconstruct the genome of PVY and other RNA viruses indicates its potential for virus detection in potato production systems, and for the study of genetic diversity of highly heterogeneous viruses such as PVY, both of which are essential to support the sustainability of the existing potato sector.

## 2. Materials and Methods

### 2.1. Plant Material

Samples (composed of six to ten leaves per potato plant) were collected during the potato growing seasons of 2017 and 2018 from plants showing symptoms ascribable to virus infection (mainly leaf mosaic, but also leaf distortion, rugosity, yellowing and crinkling). In 2017, ninety-seven plants were collected from fields hosting a diverse collection of potato germplasm (located in County Carlow and County Kilkenny). In 2018 the sampling area was expanded to commercial seed-tuber potato crops and a total of one hundred and three samples were collected from 6 different counties in the primary potato regions of Ireland (the full list of the plant samples is available in supplementary material Table S1). Upon receipt at Oak Park, all plant material was stored at −80 until use.

### 2.2. PVY Serological Tests

Two grams from each sample were weighed, placed in an extraction bag (Bioreba, Reinach, Switzerland) with 2 mL of 0.01 M phosphate-buffered saline (PBS, pH 7.4) and ground with the aid of a tissue homogenizer (Bioreba). The homogenized sample was sub-divided in two tubes for use in ELISA and nucleic acids extraction. For ELISA, 200 µL of homogenized extract were diluted with nine volumes of ELISA extraction buffer (PBS, pH 7.4 containing 0.05% Tween-20, 2% w/v PVP-40). Double-antibody sandwich ELISA [72] was performed using commercially available polyclonal/monoclonal antibodies (Pab/Mab) for PVY at the recommended dilutions. Polyclonal capture and alkaline phosphatase-conjugated antibodies for PVY were supplied by Bioreba while monoclonal antibodies specific for serotypes -N and -O/C were sourced from SASA (Science & Advice for Scottish Agriculture, Edinburgh, UK).

Nunc MaxiSorp microtiter plates (ThermoFisher Scientific, Waltham, MA, USA) were coated with capture antibodies diluted in coating buffer (15 mM Na_2_CO_3_, 35 mM NaHCO_3_; pH 9.6) and incubated at 37 °C for 4 h. Plates were washed 4 times with washing buffer (PBS pH 7.4 containing 0.05% v/v Tween 20) and 100 µL of the homogenized sample was added in duplicate to the wells of microtiter plates. Positive and negative controls obtained from Bioreba and from a collection of PVY isolates maintained at our department were also added to each plate. Following incubation overnight at 4 °C, the plates were subjected to 4 washing steps and alkaline phosphatase-conjugated antibodies were added to the wells and further incubated at 37 °C for 2 h. Plates were again washed 4 times with washing buffer prior to the addition of 100 µL of *p*-nitrophenyl phosphate, diluted to final concentration of 1 mg/mL in Substrate buffer (9.7% v/v diethanolamine, pH 9.8) and incubated at room temperature for 1 h to allow color development.

Plates were read at 405 nm using a microplate reader (Biotek, Winooski, VT, USA). Samples were considered positive when the mean OD values were greater than two times the values of the negative controls.

### 2.3. Nucleic Acids Extraction

Immediately after the initial homogenization described previously, 100 µL of each sample was added to a tube containing 9 volumes of guanidine thiocyanate grinding buffer (4.0 M guanidine thiocyanate; 0.2 M NaOAc, pH 5.2; 25 mM EDTA; 1.0 M KOAc; 2% w/v PVP-40). 500 µL was then transferred to a new tube and nucleic acids extracted by silica capture as described [73] with minor modifications, consisting of an additional washing step with 70% (v/v) ethanol and final resuspension volume reduced to 100 µL of DNase/RNase-free water.

### 2.4. RT-PCR

Reverse transcription was carried out with 2 μL of extracted RNA using RevertAid Reverse Transcriptase (ThermoFisher Scientific) as per the manufacturer’s instructions, using random hexamers (100 pmol) as reverse primers.

The cDNA (1 uL) was amplified by multiplex PCR using Taq DNA Polymerase with ThermoPol^®^ Buffer (New England Biolabs, Ipswich, MA, USA) for the detection of 5 potato viruses (PVY, PVA, PVS, PVX, PLRV) and an internal plant control (18s rRNA). The primers sequences and PCR conditions were as previously described in Du et al. [55]. The cDNA was also amplified by a second multiplex RT-PCR, targeting the main recombination sites along the PVY genome, for the identification of the PVY genotypes infecting the samples, as described in Chikh Ali et al. [56]. PCR products were separated on a 2% agarose gel stained with GelRed (Biotium, Fremont, CA, USA) and visualized in a UV transilluminator. The size of the PCR products obtained was compared to the patterns previously described in literature and with the amplicons produced by reference isolates for PVY^O^, PVY^NTN^ and PVY^NWi^ (kindly provided by Dr. Lacomme, SASA, UK). Since the PVY strain-typing RT-PCR is unable to produce distinguishable patterns for certain combinations of PVY strains co-infecting the same sample [56], in these cases we resolved the RT-PCR pattern assuming the lowest possible number of PVY strains co-infecting the same sample.

### 2.5. Illumina and ONT Sequencing

Total RNA extracts were treated with RNase-free DNase I (ThermoFisher Scientific) according to manufacturer’s instructions, individually evaluated using a Qubit fluorometer (ThermoFisher Scientific) and a Nanodrop 2000 spectrophotometer (ThermoFisher Scientific), subdivided in aliquots of 2 µg and stored at -80 before use for library construction.

#### 2.5.1. Illumina

Two micrograms of total RNA was precipitated with 2.5 volumes of ethanol and 0.1 volumes of sodium acetate and shipped to an external sequencing service provider (Macrogen Europe, Meibergdreef, Netherlands). RNA quality was assessed on an Agilent RNA 6000 Pico chip (Agilent Technologies, Santa Clara, CA, USA). Libraries preparation and sequencing were performed using the TruSeq stranded total RNA library Prep kit and the Ribozero plant rRNA depletion kit (Illumina, San Diego, CA, USA), followed by NovaSeq sequencing (paired-end reads of 150 bp in length).

#### 2.5.2. Oxford Nanopore Technologies (ONT)

Starting from the same RNA extracts used for the preparation of Illumina libraries, cDNA libraries for nanopore sequencing were also produced in order to evaluate the potential of this sequencing technology to detect and reconstruct the viral genomes of PVY and other RNA viruses infecting potatoes. First strand cDNA was synthesized with SuperScript IV Reverse Transcriptase (ThermoFisher Scientific) starting from 250 ng of total RNA. The ONT protocol only contemplates priming of the RNA with a polyT primer for reverse transcription and previous studies reported the possibility that plant viruses lacking 3′ polyA tail could potentially go undetected using the standard ONT protocol [67]. For this reason, several other methods for the preparation of cDNA libraries, including random RNA priming and the use of second strand cDNA synthesis kits [66] or using whole-transcriptome amplification kits [70] have also been described.

In order to include in our cDNA libraries any RNA virus potentially infecting the plant (even without polyA tail, e.g., poleroviruses), we evaluated the use of random hexamers tagged with a specific sequence at their 5′ end. Similarly to the protocol previously described in [74], from each sample we generated double stranded DNA libraries following the 1D Strand switching cDNA by ligation protocol (version PBCE_9009_v108_revV_18Oct2016) from Oxford Nanopore Technologies (ONT), with two different priming strategies (i) using the polyT-VN RT primer as per manufacturer’s instructions, (ii) with custom-designed random primers (5′-ACTTGCCTGTCGCTCTATCTTCNNNNNN-3′). Thermal conditions for first strand cDNA synthesis and strand switching (with minor modifications from the original ONT protocol) were: 23 °C for 10 min (only for random hexamers); 50 °C for 10 min; 55 °C for 10 min; 42 °C for 10 min. First strand cDNA was purified with Agencourt AMpure XP beads (Beckman Coulter, Brea, CA, USA) and amplified by PCR with LongAmp Taq 2X Master Mix (New England Biolabs) and ONT barcoding primers following ONT protocol. Four samples were pooled together (~75 fmol of each library) and processed as a single library with the Ligation Sequencing Kit (SQK-LSK109, ONT) according to manufacturer’s instructions. The quantity of the libraries was estimated using the Qubit Fluorometer 2.0 and the Qubit dsDNA Broad Range Assay Kit (ThermoFisher Scientific). Two libraries were produced from each plant sample selected for sequencing (one with random-primed cDNA and one with polyT-primed cDNA).

Libraries were loaded on two FLO-MIN 106D R9.4.1 flow cells: one was used for the polyT-primed libraries and one for the random hexamers-primed libraries. Sequencing was performed with a MinION (Oxford Nanopore Technologies, Oxford, UK), connected to a MinIT unit (Oxford Nanopore Technologies, UK) for twelve to seventeen hours, in order to obtain ~eight to ten million reads passing the QC check from each sequencing run. Eight samples, uniquely barcoded, were sequenced on each flow cell (FLO-MIN 106D R9.4.1) in two multiplexed sequencing runs (each sequenced library including four barcoded samples). At the start of the first sequencing run, the new flow cells had a number of nanopores available for single-strand sequencing ranging from 1400 to 1500. At the end of the first run—and after washing the flow cell—approximately 1200 nanopores were still available before the start of the second sequencing run (still considerably higher than the minimum number of 800 available nanopores that ONT set as threshold for warranty-covered replacement of the flow cell). The flow cell was then loaded again with the second library containing the remaining four multiplexed samples. Sequence reads generated were base-called in real-time using the MinKNOW software included in the ont-minit-release v. 19.06.9 (Oxford Nanopore Technologies, UK). The flow cell was washed with the Flow Cell Wash Kit between runs (Oxford Nanopore Technologies, UK) according to manufacturer’s instructions. Each flow cell was used for 2 sequencing runs, totaling eight barcoded samples, for a usage time of approximately thirty hours.

### 2.6. Analysis of Sequencing Reads

#### 2.6.1. Illumina Datasets

The raw data fastq files were checked for sequencing quality with FastQC version 0.11.8 [75] and Cutadapt v2.6 [76] was used to trim Illumina TruSeq adapters. Datasets of Illumina reads have been deposited in the Sequence Reads Archive (SRA) database at NCBI under BioProject PRJNA612026.

Subsamples of 333,334 reads (~50 million nts) were used for de novo assembly in order to achieve optimal performance of the algorithms, as the performance of de novo assembly algorithms is not optimized for very high sequencing depths [77,78,79] (>500,000 X in our case). De novo assembly was performed with CLC Genomics Workbench (Qiagen, Hilden, Germany) using default settings and CAP3 [80] was used with default parameters to merge contigs sharing nearly identical overlapping sequences (minimum 98.5% identity), thus reducing overall redundancy by collapsing shorter contigs into longer contigs and/or improving contiguity of the contigs by merging overlapping contig ends into larger contigs that were subsequently imported into CLC Genomics Workbench. Contigs were then compared for similarity against the NCBI viruses RefSeq database using BLASTn with E-value threshold set at 1 × 10^−40^ and minimum HSP (High-scoring Segment Pair) length of 1000 nts. Contigs showing similarity to plant viruses were compared against the online NCBI nr/nt database using BLASTn. The presence of suspected viral sequences was confirmed by mapping the reads to the complete viral genome sequences of the most similar complete genome viral isolates from the NCBI GenBank database, followed by visual inspection of individual mappings. Final consensus sequence of the viral genomes was obtained by mapping the reads to the complete viral genome sequence of closely related accessions from the NCBI nr database, using the “map to reference” tool in CLC Genomics Workbench with default settings (Supplementary material Figure S1). The final consensus sequences of the virus isolates sequenced in this study are publicly available in GenBank under accession numbers MT264731–MT264741.

#### 2.6.2. ONT Datasets

Raw reads obtained from each sequencing run that passed the MinKNOW quality filtering were processed with Porechop v0.2.4 [81] to trim adapters, discard chimeric reads with middle adapters and demultiplex samples with the option “require_two_barcodes” enabled and “barcode_diff ‘10.0’”. Quality check of the reads assigned to each barcode was performed with the “Fastq Control Experiment” workflow (release 3.2.2) available online through the EPI2ME platform (Oxford Nanopore Technologies, UK). The datasets of trimmed and demultiplexed ONT reads have been deposited in the Sequence Reads Archive (SRA) database at NCBI under BioProject PRJNA612026.

Each set of trimmed and demultiplexed reads was then individually mapped against the Solanum tuberosum reference nuclear, mitochondrial and chloroplast genomes [82,83] using the “map to reference” function in CLC Genomics Workbench (Qiagen) with the default settings. Unmapped reads were used for de novo assembly with Canu v1.9 [84] using; “genomeSize = 20k, rawErrorRate = 0.5, correctedErrorRate = 0.015, minReadLength = 300, minOverlapLength = 100” and the following settings as suggested for metagenomes assembly in Canu’s documentation “corOutCoverage = 10,000, corMhapSensitivity = high corMinCoverage = 0, redMemory = 32, oeaMemory = 32 and batMemory = 200” [85]. CAP3 [80] was used with default parameters to collapse and/or merge contigs sharing nearly identical overlapping sequences (minimum 98.5% identity). Contigs were then imported in CLC Genomics Workbench and compared against the NCBI viruses RefSeq database using BLASTn [86]. BLASTn results were filtered with E-value threshold set at 1 × 10^−40^ and minimum HSP length of 1000 nts. Contigs sharing significant homology to plant viruses were compared against the online NCBI nr/nt database using BLASTn. Reads were then mapped to the complete viral genome sequence of closely related accessions from the NCBI nr database using the “map to reference” tool in CLC Genomics Workbench with default settings. The final consensus sequence was extracted from the alignment (supplementary material Figure S1).

#### 2.6.3. Accuracy of ONT Sequencing

Per each barcoded sample, the base accuracy of viral reads and cumulative accuracy of de novo assembled contigs and consensus sequences obtained by a map to reference approach were assessed by comparison with the Illumina-derived consensus sequence of the viruses detected.

To estimate the base accuracy, ONT reads generated from PolyT- and random hexamers- primed libraries were mapped to the corresponding Illumina-derived virus consensus sequence in CLC Genomics Workbench, with the default settings. BAM files of the alignments were exported and processed with pysamstats [87] to extract the coverage and the number of matches, mismatches and indels at each nt position. The pileup-based statistics produced by pysamstats are available on Figshare (https://doi.org/10.6084/m9.figshare.12034617). Accuracy of the ONT reads at each nt position was reported as percentage of matches over the coverage. For clarity of display, means of similarity within 200 nt long sliding windows were calculated and plotted with R (version 3.6.1) [88] using the package ‘zoo’ [89], based on scripts available at https://github.com/loire/roumagnac2018_figs [67]

Viral contigs and consensus sequences obtained by nanopore sequencing were aligned (with the option “very accurate” enabled) to the Illumina-derived final consensus viral sequences and pairwise compared in CLC Genomics Workbench. Percent identity was calculated excluding the uncovered 5′ and 3′ ends, based on the number of matches, mismatches, insertions and deletions.

### 2.7. PVY Recombination and Phylogenetic Analyses

Recombination analysis was performed using the software as described in [14]. The complete and near complete PVY genomes obtained in this study by both Illumina and ONT sequencing were aligned by MUSCLE [90] to reference PVY genomes available in GenBank and subjected to recombination analysis using the software RDP v4.97 [91] to detect potential recombination events. The full genome sequences of at least one PVY isolate previously identified as belonging to each of the 5 non-recombinant genotypes (PVY^O^, PVY^Eu-N^, PVY^Na-N,^ PVY^C^ and PVY^O5^) were included in the analysis as potential parents. Only recombination events detected with significant support (*p* < 0.0001 after Bonferroni correction for multiple comparisons as implemented in RDP4) by all of the six analysis programs (RDP, GENECONV, Chimaera, MaxChi, Bootscan and SiScan) were considered. Detected recombination events were compared to the ones already described in literature and novel recombination sites were inferred only if distant more than 80 nucleotides from established boundaries [16].

For PVY phylogenetic analysis, the near complete viral sequences obtained in this study were aligned by MUSCLE [90] with those of the 26 accessions retrieved from GenBank, representing the non-recombinant and the “common” recombinant genetic structures of PVY [16]. GTR + G + I substitution model was determined to fit data best and was thus used for construction of a maximum likelihood phylogenetic tree in MEGA X [92] with bootstrap value of 1000. The full list of the Genbank accessions used for recombination and phylogenetic analysis is available in supplementary material (Table S2).

## 3. Results

### 3.1. PVY Serological Detection and Characterization

Serological tests were used to detect the presence/absence of PVY and to determine the serotype of PVY isolates in the two hundred potato plants sampled in different potato growing regions of Ireland during 2017 and 2018. PVY was detected by ELISA with polyclonal antibodies (Bioreba Pab, reacting with all PVY strains) in one hundred and sixty-two samples. When tested by ELISA with monoclonal antibodies, one hundred samples were typed as N-serotype based on the positive reaction with SASA-N Mab, while forty-one samples reacted with SASA-O/C antibodies, indicating an “O/C” serotype. Twenty-one samples reacted with both SASA-N and SASA-O/C Mabs, indicating mixed infections sustained by “O/C” and “N” PVY serotypes (supplementary material Table S1). Our results indicate that the “N” serotype of PVY is prevalent in the tested samples. However, since serological tests could not distinguish between different non-recombinant and recombinant PVY genotypes which exhibit the same serotype, the samples were also subjected to RT-PCR to confirm the presence of the virus and for the molecular typing of PVY isolates.

### 3.2. RT-PCR Assays for Virus Detection and PVY Strain-Typing

In order to confirm the results of the serological tests with an independent method we also performed RT-PCR for the detection of PVY, PVS, PVA, PVX and PLRV [55] in the two hundred samples. Furthermore, a second RT-PCR [56] assay was performed to better differentiate among PVY strains. Using this complementary approach, one hundred and sixty-two samples tested positive to PVY, matching the results obtained by ELISA. PVS, PVA, PVX and PLRV were detected less frequently in eighteen, eleven, nine and one sample, respectively. The incidence of PVY infections was similar in the plant material from the diverse collection of potato germplasm and from commercial seed-tuber potato crops. In contrast, PVS and PVX were only detected in the diverse germplasm collection, while PVA was predominantly found in samples from commercial seed-tuber potato crops. Only one plant was found infected by PLRV (Table 1). The detailed results for each plant sample are provided in Table S1 in supplementary material.

**Figure 1:**
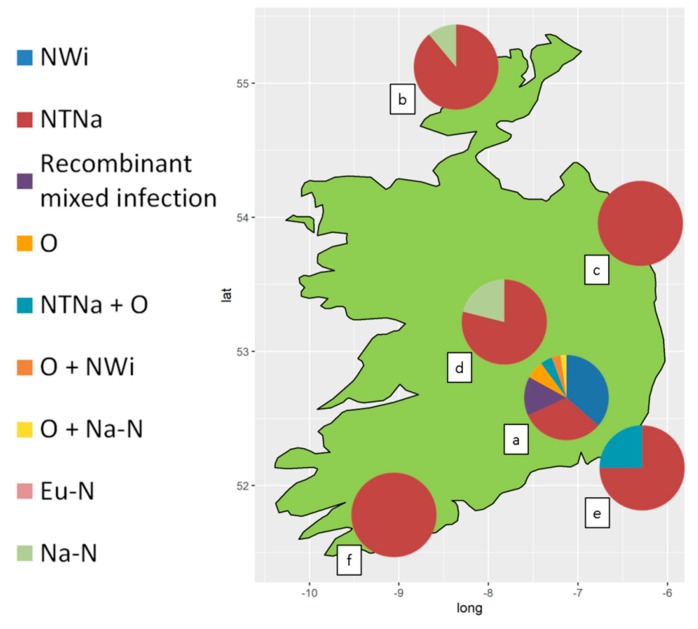
Relative incidence of PVY genotypes in different counties of the Republic of Ireland. (a) Diverse collection of potato germplasm Co. Carlow and Kilkenny (*n*= 88). Seed tuber potato crops in (b) Co. Donegal (*n*= 9); (c) Co. Louth (*n*= 34); (d) Co. Offaly (*n*= 19); (e) Co. Wexford (*n*= 8); (f) County Cork (*n*= 4).

All samples positive to PVY produced specific bands when amplified with the multiplex RT-PCR protocol for strain-typing. Overall, recombinant genotypes accounted for the vast majority of the PVY infections detected. Based on the pattern of PCR products, sixty-four samples were typed as singularly infected by PVY^NTNa^, thirty-two by PVY^NWi^, six by PVY^O^, five by PVY^Na-N^ and one by PVY^Eu-N^. Patterns attributed to mixed infections of PVY^NTNa^+PVY^NWi^ (thirteen samples), PVY^NTNa^+PVY^O^ (six samples) PVY^O^+PVY^NWi^ (three samples) and PVY^O^+PVY^Na-N^ (one sample) were also observed. Thirteen samples produced amplicons ascribable to co-infection sustained by multiple combinations of recombinant strains, but the RT-PCR assay couldn’t resolve the PVY genotypes involved (Table 2). In plant samples from the diverse germplasm collection, PVY^NWi^ and PVY^NTNa^ were the most commonly detected genotypes. PVY^NWi^ found, in singular or mixed infection with other PVY genotypes, in 39.8% (35/88) and 36.4% (32/88) of the tested samples, respectively. In contrast, PVY^NWi^ was not detected in commercial seed-tuber crops, where 89.2% (66/74) of the PVY infections were exclusively sustained by PVY^NTNa^ (Table 2). The relative incidence of PVY genotypes and mixed infections in the different regions of Ireland is shown in Figure 1. Detailed results per each sample are listed in supplementary material (Table S1).

The results of the RT-PCR analyses are in agreement with the serological tests and provided more detailed information on the PVY genotypes infecting the plant samples of this study. In fact, recombinant PVY^NTNa^ and non-recombinant PVY^Eu-N^ and PVY^Na-N^ all exhibited a “N” serotype and could not be distinguished with ELISA tests, reacting with SASA-N Mab. Similarly, both non-recombinant PVY^O^ and recombinant PVY^NWi^ reacted with the SASA-O/C Mab in ELISA tests. With the information obtained from serological and molecular tests, we selected eight samples infected with different PVY genotypes and/or infected with other RNA viruses for Next Generation Sequencing and determination of the whole genome sequences.

### 3.3. Illumina Sequencing

Since the strain-typing RT-PCR assay only targets the most common recombination sites and can potentially mistype “rare” and newly described genotypes [16], eight samples showing single or mixed infections sustained by different PVY genotypes, PVX, PVS and PLRV (as revealed by the RT-PCR assays) were selected and subjected to Illumina sequencing technology in order to reconstruct the whole genome viral sequences. The samples included all of the five PVY genotypes previously detected by RT-PCR (Table 3). The RNA extracts used for library preparation exhibited RIN (RNA Integrity Number) values ranging from 2.1 to 5.7 (supplementary material Table S3), indicating variable degrees of RNA degradation, which possibly occurred between the collection in the field and the arrival of the sample at our laboratory or during freezing/thawing processes before RNA extraction. Nevertheless, more than 40 M single end reads were generated from each sample (ranging from 46.26 M to 60.38 M) (Table 3). After trimming of adapters, a subsample of 333,334 reads per sample was used for de novo assembly with CLC Genomics Workbench and CAP3. In every sample but P221, we were able to successfully reconstruct the complete or near complete genomes of all viruses known to be present in the samples based on our serological tests and RT-PCR assays (Table 3). BLASTn analysis of the de novo assembled contigs against the NBCI virus RefSeq database showed a single contig representing the near complete genome of every virus previously known to infect the sample, including the complete coding sequence and only missing the last nucleotides at the non-protein-coding 5′ and/or 3′ end of the genome (Table 3). In the case of sample P221 (mixed infection of PVY strains), four contigs sharing high homology (BLASTn E-value ≤ 1 × 10^−40^ and HSP ≥ 1000 nts) with PVY were assembled, that ranged in length from 2217 to 3490 nucleotides.

After BLASTn searches against the online NCBI nr/nt database, two contigs showed the highest identity to PVY^NWi^ accessions, while the remaining two showed the highest identity to PVY^NTNa^ accessions (Table 3).

BLASTn searches against the online NCBI nr/nt database allowed us to identify the closest full genome sequence available in GenBank for every assembled viral contig. For every identified virus/virus variant, the consensus sequence was extracted from the Illumina read mappings in order to obtain a corrected consensus genome. The whole genome sequences obtained by a mapping to reference approach were longer than the de novo generated contigs and often completely covered the complete viral genome, including the non-protein-coding 5′ and 3′ ends (Table 3).

Illumina sequencing allowed the reconstruction of the complete, or near-complete, genome of the viruses previously known to have infected the eight samples. Even though the complete genome of the two PVY genotypes co-infecting sample P221 could not be de novo assembled, two contigs per each genotype, covering together more than 50% of the PVY genome, were assembled and subjected to BLASTn analysis to identify the most closely related PVY accessions deposited in GenBank. Mapping the Illumina reads to their closest PVY full genome accession allowed us to obtain a consensus sequence representing the complete genome sequence of both PVY genotypes. For all of the viruses detected, the consensus sequence extracted from the read mappings was used as a gold standard to compare the performance and accuracy of ONT’s MinION sequencing. The final consensus sequences of the virus isolates sequenced in this study are publicly available in GenBank under accession numbers MT264731–MT264741.

### 3.4. ONT Sequencing

**Figure 2:**
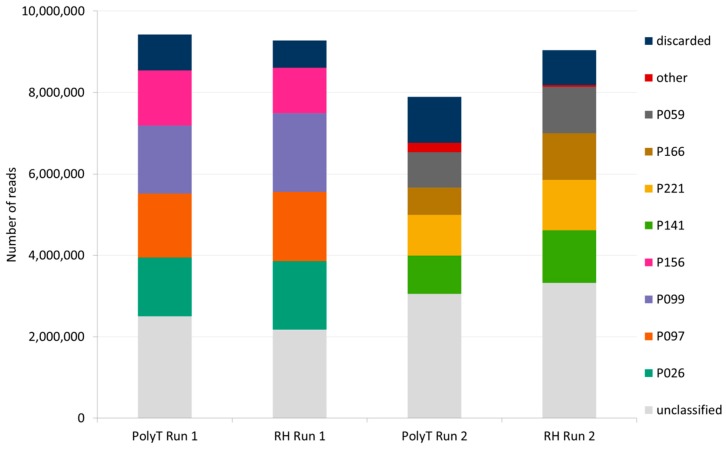
Sequencing yield in number of Oxford Nanopore Technologies (ONT) reads generated from each sequencing run, which passed the internal MinKnow filtering parameters (pass) and were further analyzed by Porechop for adapters trimming and barcode demultiplexing. Stacked bars are color coded according to Porechop analysis. A washing step was performed between two consecutive runs on the same flow cell (PolyT Run 1–2 and RH Run 1–2).

PolyT-primed and random-primed libraries, each one containing four barcoded samples, were sequenced in two runs with a MinION sequencer using two flow cells: one for PolyT-primed libraries and one for random-primed libraries (Figure 2).

#### 3.4.1. Nanopore Sequencing Throughput

The total number of reads generated in a sequencing run (including 4 barcoded samples) and passing the internal MinKnow quality filtering ranged from 7.9 M to 9.46 M (Figure 2, supplementary material Table S4). After trimming of adapters and demultiplexing, the number or reads ranged between 668,443 and 1,934,615 per each barcoded sample. Though both sequencing runs on each flow cell were able to produce ~ eight to ten million reads, the first sequencing run on each flow cell produced a slightly higher number of reads than the second run and a lower number of unclassified reads. (Figure 2, supplementary material Table S4).

#### 3.4.2. De Novo Assembly and Virus Detection

De novo assembly was independently performed on two datasets of reads per each plant (from polyT-primed and random-primed cDNA, respectively) and the resulting contigs were then compared to the NCBI virus RefSeq databases with BLASTn in order to identify contigs of viral origin. After removing reads originating from the host plant, the number of reads available for de novo assembly ranged from 13,637 to 142,641 reads per sample (supplementary material Table S4). Assembly of the reads was performed using Canu and CAP3, with the minimum identity in an alignment of two reads set at 98.5% in order to avoid the generation of “hybrid” assemblies containing reads belonging to different strains of the same virus potentially co-infecting the sample. In both polyT- and random hexamers-primed cDNA samples, all of the viruses previously known to infect the samples were detected after de novo assembly and a BLASTn search of the contigs against the NCBI Viral RefSeq database (Table 4). Interestingly, in sample P166 contigs showing high homology to PLRV (BLASTn E-value < 1 × 10^−40^) were assembled from both random-primed and polyT- primed cDNA datasets. (Table 4). De novo assembly of reads generated from random hexamers-primed libraries resulted in contigs representing the near complete viral genomes for all of the viruses detected. Also in sample P221, previously known to be infected by different strains of PVY, two near complete genome contigs were assembled. BLASTn search of the contigs against the online NCBI nr/nt database revealed the first shared high sequence homology with PVY^NTNa^ and the second contig shared high sequence homology with PVY^NWi^. Other than PVY, near complete genomes were assembled also for PVS and PVX, co-infecting sample P059 together with PVY. Ten contigs with high homology (BLASTn E-value ≤ 1 × 10^−40^ and HSP ≥ 1000nts) to PVX were assembled, all of them sharing the highest identity to PVY isolate JAL-2 (GenBank accession KR605396) and with the longest one covering the complete viral genome (Table 4). A contig covering >89% of the genome of PLRV, belonging to the family *Luteoviridae* and lacking a polyA tail at the 3′ of its genome, was detected in sample P166. Similarly, de novo assembly of reads from polyT-primed libraries produced contigs of comparable length to the ones generated from random hexamers-primed libraries, with the only exceptions of samples P166 and P221. In sample P221 only one near complete contig representing PVY^NTNa^ was assembled, while three contigs were generated for the second PVY genotype (PVY^NWi^) infecting the sample. The longest PVY^NWi^ contig covered ~74.5% of the viral genome. However, taken together the three PVY^NWi^ contigs covered ~98% of the virus genome. In sample P166, the longest contig matching PLRV reference genome covered ~63.4% of the genome. Even though a single contig spanning the near complete genome of PLRV (in sample P166) and PVY^NWi^ (in sample P221) could not be assembled from polyT- primed cDNA samples, shorter virus- and strain-specific contigs were assembled, allowing the detection of both PVY^NWi^ and PLRV (Table 4). The sequences of the de novo assembled viral contigs are available on Figshare (https://doi.org/10.6084/m9.figshare.12034254).

#### 3.4.3. Map to Reference

In order to confirm the presence of the suspected viral sequences in the ONT datasets, the reads were individually mapped to the complete genome sequences of the most similar viral isolates from the NCBI GenBank database (as identified by BLASTn searches of the de novo assembled contigs against the online NCBI nr/nt database), followed by visual inspection of individual mappings (Table 4). From each alignment we extracted the consensus sequence to obtain a corrected consensus genome sequence for every virus/virus-variant detected in our samples. Inspecting the alignments, we could observe that a higher percentage of reads from polyT-primed datasets mapped to the reference sequence in comparison to the percentage of reads from random hexamers-primed datasets, resulting in higher average sequencing depth of the viruses. The only exceptions to this were samples P166 and P059 (Table 4, supplementary material Table S4). Although higher, sequencing depth in polyT-primed libraries was less homogeneously distributed along the viral genome, with peaks at the 3′ end and in several other segments of the genome (supplementary material Figure S2), suggesting non-specific annealing of the polyT primer during reverse transcription of the RNA. Furthermore, non-specific binding of the polyT primer during reverse transcription could also explain the generation of PLRV-specific reads in the polyT-primed dataset for sample P166 that were successfully mapped to the non-polyadenylated PLRV reference sequence.

Despite the difference in viral sequencing depth between polyT- and random hexamers-primed datasets, there was no sizable difference in length of the final consensus sequence. A final consensus sequence representing the complete or near complete genome was obtained for each virus/virus variant. These ranged in length from 9682 to 9697 nts for PVY, 5881 to 5882 nts for PLRV, 6433 to 6434 nts for PVX and 8497 to 8498 nts for PVS (Table 4). Overall, mapping the ONT reads to reference virus sequences confirmed the presence of PVY, PLRV, PVX and PVS viral reads in both random-primed and polyT-primed datasets and allowed us to obtain a corrected consensus genome sequence for every virus/virus variant detected in our samples. Although we did not expect to detect and reconstruct the genome of PLRV from polyT-primed cDNA samples, the virus was detected and its complete genome was extracted from the read mapping, probably because of non-specific annealing of the polyT primer during reverse transcription. The consensus sequences extracted from the alignments of ONT reads to the reference GenBank accessions are available on Figshare (https://doi.org/10.6084/m9.figshare.12034200).

#### 3.4.4. Accuracy of ONT Sequencing

In order to compare the performance of polyT-primed and random-primed cDNA sequencing, the base accuracy of viral reads and cumulative accuracy of de novo assembled contigs and consensus sequences obtained from each dataset were assessed with the Illumina-derived viral consensus sequences.

**Figure 3:**
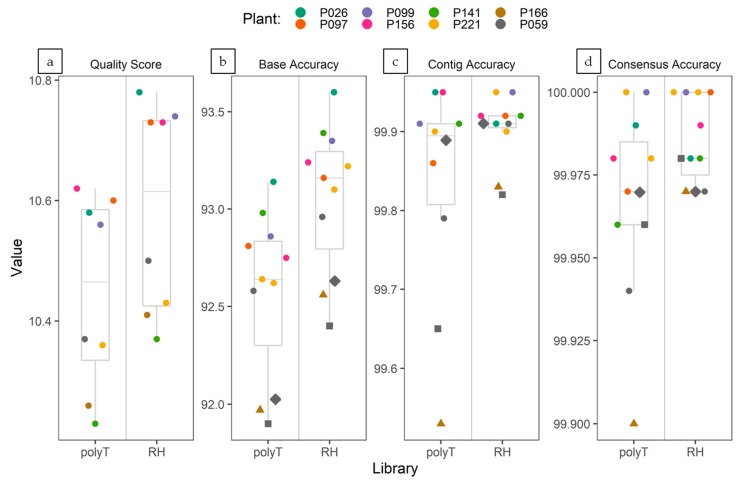
Box plots depicting quality score (**a**) and accuracy of ONT reads (**b**), contigs (**c**) and consensus sequences (**d**) generated from the PolyT- and random hexamers-primed libraries. The lower and upper hinges correspond to the first and third quartiles; the upper whisker extends from the hinge to the largest value no further than 1.5 * IQR from the hinge (where IQR is the inter-quartile range). The lower whisker extends from the hinge to the smallest value at most 1.5 * IQR of the hinge. For clarity of display, the y axis on each graph was scaled accordingly. (**a**) Quality score as calculated by the EPI2ME online platform. (**b**) Accuracy of ONT virus-related reads as percentage of matches over the coverage at each nt position. (**c**) Percent identity of ONT-derived viral contigs compared to the correspondent Illumina consensus. (**d**) Percent identity of ONT-derived viral consensus sequences compared to the correspondent Illumina consensus. In 3b, 3c and 3d dots refer to PVY, triangles to PLRV, squares to PVS and diamonds to PVX.

The average quality (q-score) of the ONT reads generated from each barcoded cDNA sample was calculated with the “Fastq Control Experiment” workflow (release 3.2.2) available online through the EPI2ME platform and ranged from 10.3 to 10.8. There was minimal difference in the quality of reads generated by the flow cells loaded with either PolyT-primed and random hexamers-primed libraries. Since two different flow cells were used, it could be explained by the normal variability between flow cells. In both PolyT- and random hexamers-primed flow cells, the library loaded in the first sequencing run generated reads exhibiting slightly higher q-score than the library loaded in the second run (following washing of the flow cell). Despite the slight difference, average q-score of the reads in every library was well above the threshold for low quality reads indicated by the EPI2ME platform (q-score= 7) (Figure 3a). Base accuracy of ONT virus-related reads, as estimated by mapping to the Illumina-derived virus consensus sequences, ranged from 91.90% to 93.14% for PolyT-primed libraries, and from 92.40% to 93.60% for random hexamers-primed libraries (Figure 3b). For both library types, mapping of the reads to the reference Illumina-derived viral consensus showed an even distribution of sequencing errors (gaps and mismatches) across the genome (supplementary material Figure S3). The consensus sequences obtained by a mapping-based approach of the ONT reads shared higher nt identity % to the Illumina-derived consensus than the de novo assembled contigs, nevertheless both de novo assembled and consensus sequences obtained from ONT sequencing always showed nt identity >99.50% (≥99.90% for the consensus sequences) when compared to the Illumina-derived viral consensus sequence (Figure 3c,d).

Overall, although the base accuracy of single ONT virus-related reads was estimated to range between 91.90% and 93.60%, the accuracy of de novo assembled contigs and alignment-based consensus sequences was >99.50% and ≥99.90%, respectively. The degree of accuracy is consistent with previous reports [67,71,93,94]. Hence, when evaluating the base accuracy of ONT reads and the cumulative accuracy of contigs and consensus sequences generated from random-primed cDNA libraries, these were of at least comparable quality compared to the ones produced with the standard ONT protocol.

### 3.5. PVY Recombination and Phylogenetic Analyses

**Figure 4:**
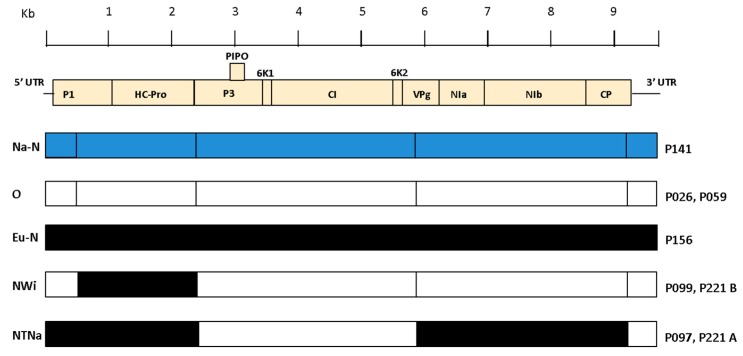
Schematic representation of the genetic structures of PVY isolates sequenced in this study. A different color (blue, white and black) is assigned to each non-recombinant genotype, recombinant genotypes are colored accordingly. Names of the PVY genotypes are listed on the left side of Figure, while the corresponding isolates are listed on the right. The breakpoints in the alignment related to the identified recombinant genotypes are marked with vertical black bars. PVY genome (~9.7 Kb) is drawn at the top, with cistrons represented as rectangles with the corresponding protein names.

Recombination analysis based on aligned whole genome sequences is essential in order to be certain of the true genetic structure of individual isolates and avoid mistakes in genotype classification of the PVY variants [16]. Analysis of the PVY genomes obtained in this study conducted with RDP4 [91] identified four breakpoints in the alignment, corresponding to previously known recombination sites already described in literature [14,16]. Illumina-derived viral consensus sequences and ONT-derived contigs and consensus produced the same results for all samples. Based on the breakpoint positions in the alignment and the parental genotypes (Table 5), P097 was typed as PVY^NTNa^ and P099 as PVY^NWi^, while P221 was determined to be infected by both PVY^NTNa^ and PVY^NWi^ strains (Figure 4). The PVY genomes included in the alignment were divided into 5 sections, delimited by the four recombination sites previously detected (nt positions 3′ end–500, 501–2390, 2391–5850, 5851–9200 and 9201–5′ end) and a phylogenetic tree was constructed per each PVY genome section (supplementary material Figure S4). Individual phylogenetic analysis of every PVY genome section was consistent with the results of the recombination analysis conducted with RDP4.

**Figure 5:**
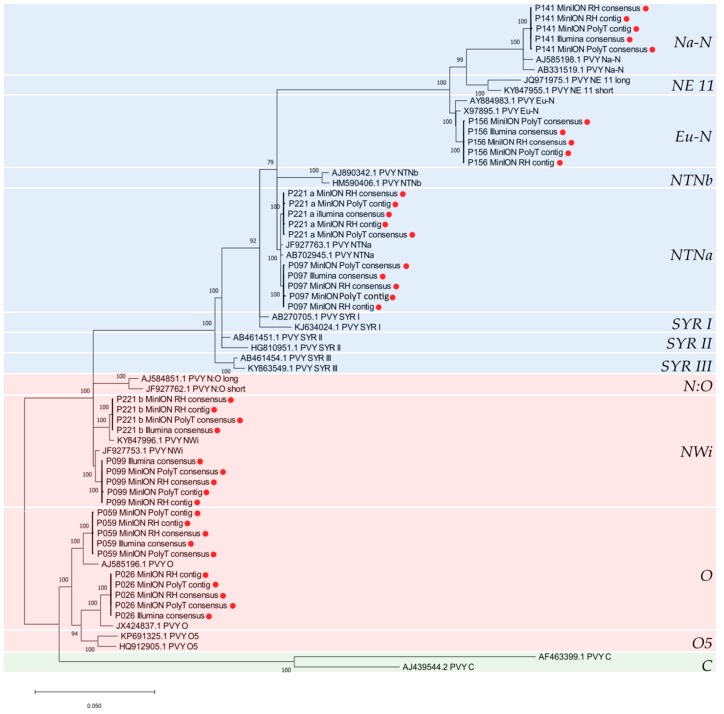
Maximum likelihood phylogenetic tree obtained from the PVY sequences obtained in this study (marked with a red dot) and other 26 PVY genomes retrieved from GenBank. Branch lengths indicate the number of substitutions per site. Only bootstrap values higher than 70% are shown. Serological groups are indicated with different background colors (green for C serotype, red for O serotype and blue for N serotype). Boxes indicate different genotypes of the virus; name of the genotype is reported on the right side of the tree.

The phylogenetic tree generated from the alignment of the whole PVY genomes isolates is shown in Figure 5. Isolates P026, P156, P141 and P059, previously typed as non-recombinant based on the results of the RT-PCR assay, clustered in the PVY^O^ (P026 and P059), PVY^Eu-N^ (P156) and PVY^Na-N^ (P141) phylogroups. Isolates P099 and P221b grouped together with Genbank accessions JF927753 and KY847996, previously typed as PVY^NWi^, while P097 and P221a clustered with Genbank accessions JF927763 and AB702645, previously typed as PVY^NTNa^ [14]. Per each PVY isolate, the near full-length Illumina final consensus and ONT-derived contigs and consensus were included in the analysis and clustered identically in the phylogenetic tree (Figure 5).

With recombination and phylogenetic analyses of the PVY genomes we were able to establish their genetic structure and therefore definitively assign the PVY isolates to their respective genotypes, including the two PVY variants co-infecting sample P221, that could not be conclusively typed with the conventional RT-PCR assay.

## 4. Discussion

PVY is considered the most important virus pathogen threatening potato production worldwide. Despite having a wide host range, host plants of PVY in most potato production regions are annual and the virus needs to be re-introduced in the field every year [24]. Planting of infected seed-tubers and infected tubers remaining from the previous harvest are the main source of initial inoculum. Consequently, certification of the propagation material plays an important role in controlling the spread of PVY in potato crops. Visual inspection of seed-tuber production crops during the growing season usually enables virus incidence to be estimated and for roguing of plants showing foliar symptoms ascribable to viral pathogens. However, the emergence and spread of recombinant PVY strains during the last three decades poses a challenge to the certification process and therefore to the control of PVY.

The diversity of PVY isolates is reflected in a wide range of symptomatology induced in infected potato plants. For instance, the ordinary strain PVY^O^, once predominant in potato crops, induces a severe mosaic in many potato cultivars, while many of the emerging recombinant PVY strains, especially the widely distributed PVY^NTN^, PVY^N-Wi^ and PVY^N:O^ often induce mild or transient foliar symptoms in many potato cultivars [33,40,95,96], making them more difficult to detect during visual inspection in the field. Accurate detection and characterization of the PVY isolates circulating in potato crops is therefore essential. In the current study, we investigated PVY diversity in two hundred symptomatic potato plants collected from seed-tuber crops and potato germplasm collections and evaluated the use of ONT sequencing for the detection and molecular characterization of the virus.

As revealed by ELISA, both PVY^N^ and PVY^O^ serotypes are present, with the PVY^N^ serotype being dominant. RT-PCR analyses for strain-typing of the PVY isolates indicated that, within both serotypes, recombinant genotypes accounted for the vast majority of PVY infections. More than 90% of the PVY infections detected were sustained by recombinant strains (singularly or in mixed infection with other strains). Within non-recombinant PVY genotypes, representing a minority of the PVY isolates, are PVY^O^, PVY^Na-N^ and PVY^Eu-N^. Although the ^Eu-^ and ^NA-^ prefixes (for “North American” and “European”, respectively) identify distinct PVY^N^ genotypes, they are no longer indicative of the geographical origin of PVY isolates, as PVY^Na-N^ has been found in Europe as well and vice versa [37,54,97,98]. To the best of our knowledge, this is the first report of the North-American N genotype of PVY (PVY^Na-N^) in Ireland. However, we cannot exclude that this genotype was already present in Ireland but went un-noticed, since PVY^Na-N^ cannot be distinguished from other PVY isolates exhibiting PVY^N^ serotype by ELISA tests [54,98]. The genetic diversity of PVY isolates circulating in seed tuber crops in Ireland, with PVY^NTNa^ as the predominant genotype, is not dissimilar from recent reports from Scotland [37]. Considering its association with the induction of PTNRD symptoms on tubers and its widespread diffusion, PVY^NTNa^ represents a potential threat to PTNRD-susceptible potato cultivars. The relevant presence of PVY^NWi^ in the diverse germplasm collection (as opposed to seed-tuber crops collected throughout the country) may be partially explained by the different origin of the plant material. This diverse collection of potato germplasm includes plant material imported from mainland Europe, where PVY^NWi^ is more widely distributed [46,99]. PVY isolates detected in thirteen plant samples, presenting a mixed infection sustained by at least one recombinant PVY genotype, could not be typed with the multiplex RT-PCR assay [56]. The assay was able to detect mixed infections but could not unambiguously identify PVY^NTNa^ and PVY^NWi^ genotypes in mixed infections.

The inability of small RNA sequencing approaches to fully and reliably reconstruct from mixed infections the genome sequences of two (or more) strains or genetic variants of the same virus when they share high sequence identity has already been pointed out [100]. The potential use of ONT sequencing to resolve and fully reconstruct viral genomes from non-clonal viral populations has recently been suggested [67,100]. Here, we report the successful de novo reconstruction of two distinct recombinant strains of PVY co-infecting the same plant sample by Canu and CAP3 assembly of ONT’s MinION reads. It is likely that the longer read length achievable by ONT sequencing enabled the de novo assembly of distinct contigs for each virus variant, thereby avoiding the assembly of chimeric contigs for highly similar portions of the genomes. The two PVY variants involved in the mixed infection could also be identified by BLASTn analysis of the shorter contigs produced by Illumina, allowing the reconstruction of the complete genome of both strains by a map to reference approach. However, the ability to generate distinct, de novoassembled genomes of both PVY variants from the MinION reads could potentially facilitate in the discovery and molecular characterization of novel virus variants absent from the NCBI GenBank database. Random priming of the RNA in ONT library preparation resulted in reads of comparable quality and length to the standard protocol but mapping more homogenously along the viral genomes. While the standard ONT polyT-primed library preparation enriches the library for polyadenylated RNA sequences, it has been speculated that it could fail to include non-polyadenylated RNA viruses [67]. Conversely, random priming of total RNA is expected to include any potential virus present in the sample [101], even if at a lower sequencing depth because of the highly abundant plant ribosomal RNA present in the total RNA extract. In our experience PLRV (lacking a polyA tail at the 3′ end of its genome) could also be detected in polyT-primed preparations, possibly because of non-specific annealing of the polyT primer during reverse transcription.

In order to definitively assign the PVY variants detected in this study to a PVY genotype, we aligned the PVY genomes to a set of reference sequences available in GenBank and conducted recombination and phylogenetic analysis. This enabled the identification of strains PVY^NTNa^ and PVY^NWi^ in one of the mixed infections that could not be typed by the conventional RT-PCR assay. Furthermore, it confirmed infections sustained by PVY^O^, PVY^NTNA^, PVY^NWi^, PVY^Eu-N^ and PVY^Na-N^ in the samples sequenced. In addition to PVY, we also assembled near complete genomes of PVX, PVS and PLRV using both Illumina and ONT sequencing. Genotyping based on the analysis of ONT-generated viral genomes, both by de novo assembly or map to reference approaches, exactly matched the genotyping results obtained with Illumina-derived genomes.

The routine implementation of next generation sequencing (including Illumina and ONT technologies) in plant pathology diagnostic laboratories still faces challenges. These include harmonization of wet lab and computational protocols and validation of NGS assays as reported in EPPO PM7/98. A number of review and opinion papers discussing the application of next generation sequencing to plant pathogen diagnostics have been published in recent years [101,102,103,104,105,106,107]. For example, it has been pointed out how different nucleic acids extraction protocols and their ability to include and/or enrich for viral nucleic acids, may affect the ability of the NGS assay to detect the whole range of potential viral pathogens [101,107]. Another important aspect to be addressed is the analytical sensitivity of NGS tests and the determination of a threshold to avoid false positive and false negative results [101]. Compared to conventional diagnostic tests, the non-targeted nature and the lack of controls for potentially any pathogen that could be detected in a sample by next generation sequencing is one of the key aspects to address in order to enable effective monitoring of the diagnostic test’s performance. Therefore, instead of traditional validation, there has been a greater focus on the application of internal quality control [106] and different approaches have been explored, such as using spikes of endornavirus-infected leaf discs [108] and synthetic RNA standards [109]. However, there is enormous potential in having the ability to detect and sequence potentially any virus and viroid present in a sample in a single assay and it is clear that NGS is becoming an increasingly important and additional tool in plant virus diagnostics [106].

The MinION is an inexpensive and portable sequencer that can be used in any laboratory without a need for special equipment, and in some cases its use in-field has been demonstrated [69]. This avoids the need to ship samples to external sequencing service providers and consequently reduces the time from sampling to identification

The results we described from the MinION sequencing of RNA viruses infecting potatoes provide new information on the use of this device for the detection, genome sequencing and genotyping of closely related virus strains, and its ability to distinguish and assembly the genomes of different virus strains in non-clonal viral populations. In our experience, the ability of ONT’s MinION sequencing to detect and reconstruct the genome of RNA viruses was not hindered by degradation of the total RNA extracts used for library construction. RNA degradation possibly happened between samples collection and receipt at our laboratory, or because of freezing/thawing processes before RNA extraction. In our case, samples collected from seed-tuber crops were posted to our lab by standard post without refrigeration.

There are still a range of aspects to be investigated and addressed, such as the potential use of different nucleic acids preparations (e.g., dsRNA and virion-associated nucleic acids) for generation of virus-enriched sequencing libraries, the ability of the sequencing libraries to capture viruses belonging to different groups of the Baltimore viral classification [110], and determination of the limit of detection. DNA extraction and library preparation for MinION sequencing in the field has recently been reported [69]; encouragingly authors were able to sequence and detect several DNA viruses and plant pests within three hours of sample collection. Advances in automated sample preparation that enable samples to be prepared and sequenced directly in a crop field, combined with even smaller and more portable devices and efficient data processing [111] will further accelerate the deployment of in-field viral diagnostics via sequencing.

Other technologies based on nano-sensors are also being developed for rapid in-field diagnosis of targeted plant pathogens [112,113,114,115]. These rapid assays could be used directly by farmers and field inspectors with minimal training, thereby facilitating the binary diagnosis of plant viruses without the need of trained staff for assay execution and avoiding the need to ship plant samples to diagnostic laboratories. However, the non-targeted nature of the ONT’s MinION sequencing and its ability to reconstruct whole virus genomes will facilitate the detection and sequencing of potentially any virus in a single assay, including novel and not previously described viruses or virus genotypes. Moreover, its portability and the possibility to analyze sequencing data directly in the field makes it a suitable tool to investigate the genetic diversity of plant viral pathogens in remote locations.

## 5. Conclusions

The presence of PVY recombinant isolates has been reported in the Republic of Ireland [53] however, information on the relative incidence of PVY recombinants in potato crops was missing. In the current study, we have characterized Irish PVY isolates using conventional serological and molecular assays and found that recombinant genotypes were prevalent, including the PVY^NTNa^ genotype, which was predominant.

This is the first report describing the use of ONT sequencing to identify and genotype RNA viruses in potato. In particular, we show the ability to (i) detect both polyadenylated and non-polydenylated viruses, and (ii) to distinguish and correctly identify non-recombinant and recombinant strains of potato virus Y in a single assay. In conclusion, these results demonstrate that it is possible to identify RNA viruses in potato samples using portable nanopore technology, which provides a novel platform to support the management of appropriate crop hygiene practices in commercial potato production systems.

## Figures and Tables

**Table 1:** Viruses detected by multiplex RT-PCR in plant samples collected from the diverse collection of potato germplasm and from seed-tuber potato crops. Samples were tested as described in Du et al. [55] for the presence of potato virus Y (PVY), potato virus S (PVS), potato virus X (PVX), potato leafroll virus (PLRV) and potato virus A (PVA).

	PVY (Positive/Tested)	PVS (Positive/Tested)	PVX (Positive/Tested)	PLRV (Positive/Tested)	PVA (Positive/Tested)
Germplasm collection	74/90	0/90	0/90	1/90	10/90
Seed-tuber Potato crops	88/110	18/110	9/110	0/110	1/110
Total	162/200	18/200	9/200	1/200	11/200

**Table 2:** Properties of PVY isolates infecting plant samples collected from the germplasm collection and from seed-tuber potato crops, as determined by DAS-ELISA with monoclonal antibodies and by multiplex RT-PCR for differentiation PVY strain [56].

PVY Genotype	NTNa	NWi	Recombinant Mixed Infection ^1^	O	NTNa+O	Na-N	O+NWi	O+Na-N	Eu-N
RT-PCR pattern (bp)	441+633 +1307	441+853	441+633 +853+1307	532+853	441+532 +633+853 +1307	1307	441+532 +853	532+853 +1307	398+633 +1307
**Serotype**	**N**	**O/C**	**N + O/C**	**O/C**	**N + O/C**	**N**	**O/C**	**N + O/C**	**N**
Germplasm collection	28/88	32/88	13/88	6/88	4/88	0/88	3/88	2/88	0/88
Seed-tuber Potato crops	66/74	0/74	0/7	0/74	2/74	5/74	0/74	0/74	1/74
Total	94/162	32/162	13/162	6/162	6/162	5/162	3/162	2/162	1/162

^1^ NTNa+NWi or NTNa+N:O or NTNb+N:O or N:O+NaN.

**Table 3:** Summary of Illumina sequencing and assembling statistics.

Sample ID	RT-PCR Virus	RT-PCR PVY Genotype	Total Reads	Virus Contigs/HSP Length (nt) ^1^	NCBI BLASTn Accession ^2^ (Length nt)	Consensus Length ^3^	Average Sequencing Depth	Assigned PVY Genotype ^4^
P026	PVY	O	60.38 M	1/9694	PVY JX424837 (9699)	9699	175,369 ×	O
P097	PVY	NTNa	54.12 M	1/9672	PVY JF927763 (9701)	9694	31,972 ×	NTNa
P099	PVY	NWi	52.89 M	1/9683	PVY JF927753 (9697)	9697	51,748 ×	NWi
P156	PVY	Eu-N	53.56 M	1/9687	PVY X97895 (9701)	9701	569,071 ×	Eu-N
P141	PVY	Na-N	48.48 M	1/9690	PVY AB331517 (9701)	9701	593,890 ×	Na-N
P221	PVY	recombinant mixed infection	52.61 M	a. 2/2217–3490 b. 2/2217–3453	PVY JF927763 (9701) PVY JF927754 (9697)	9701 9697	124,208 × 95,512 ×	NTNa NWi
P166	PLRV		54.28 M	1/5868	PLRV AY138970 (5884)	5884	599,408 ×	
P059	PVY PVX PVS	O	46.26 M	1/9674 1/6427 1/8463	PVY MH795851 (9700) PVX KR605396 (6435) PVS MF418030 (8499)	9687 6435 8499	6384 × 556,895 × 79,253 ×	O

^1^ contigs showing E-value ≤ 1 × 10^−40^ and HSP ≥ 1000 nts after BLASTn search against the NCBI Viral RefSeq database. ^2^ closely related complete genome sequence(s) identified after BLASTn search of the viral contigs against the online NCBI nr/nt database. ^3^ after mapping reads to the reference sequence (length of consensus excluding the polyA tail). ^4^ based on closely related PVY complete genome sequences identified after BLASTn search against the online NCBI nr/nt database.

**Table 4:** Summary of nanopore sequencing and assembling statistics.

Sample ID	Library	No. of Virus Contigs/ HSP Length (nt) ^1^	Map to Reference (NCBI Accession) ^2^	Consensus Length ^3^	Average Sequencing Depth
P026	PolyT	1/9562	PVY (JX424837)	9684 nt	1027.28 ×
P097	PolyT	1/9685	PVY (JF927763)	9697 nt	478.40 ×
P099	PolyT	1/9680	PVY (JF927753)	9692 nt	946.96 ×
P156	PolyT	1/9693	PVY (X97895)	9697 nt	1855.30 ×
P141	PolyT	1/9678	PVY (AB331517)	9697 nt	1426.61 ×
P221	PolyT	a. 1/9671 b. 4/1314–7239	PVY (JF927763) PVY (JF927754)	9698 nt 9688 nt	501.54 × 734.40 ×
P166	PolyT	2/2427–3796	PLRV (AY138970)	5882 nt	81.49 ×
P059	PolyT	3/1019–8478 11/1170–6434 1/9440	PVS (MF418030) PVX (KR605396) PVY (MH795851)	8498 nt 6434 nt 9682 nt	832.57 × 6741.76 × 81.10 ×
P026	RH	1/9676	PVY (JX424837)	9682 nt	537.14 ×
P097	RH	1/9675	PVY (JF927763)	9691 nt	271.69 ×
P099	RH	1/9668	PVY (JF927753)	9688 nt	623.69 ×
P156	RH	1/9690	PVY (X97895)	9694 nt	962.31 ×
P141	RH	1/9685	PVY (AB331517)	9689 nt	1196.59 ×
P221	RH	a. 1/9684 b. 1/9625	PVY (JF927763) PVY (JF927754)	9699 nt 9688 nt	399.30 × 553.74 ×
P166	RH	1/5338	PLRV (AY138970)	5881 nt	155.26 ×
P059	RH	1/8476 10/1244–6435 1/9622	PVS (MF418030) PVX (KR605396) PVY (MH795851)	8497 nt 6433 nt 9689 nt	1144.05 × 6614.26 × 71.62 ×

^1^ contigs showing E-value ≤ 1 × 10^−40^ and HSP ≥ 1000 nts after BLASTn search against the NCBI Viral RefSeq database. ^2^ Closely related complete genome sequence(s) identified after BLASTn search of the contigs against the online NCBI nr/nt database. ^3^ after mapping reads to the reference sequence (length of consensus excluding the polyA tail).

**Table 5:** Recombination events identified in the sequenced PVY isolates.

PVY Sequence	Breakpoints Positions in Alignment	Established Breakpoints Previously Reported in Literature [16]	Non-Recombinant Parents	Genotype
P026	-		-	O
P097	2419, 5818, 9190	2390, 5850, 9200	N, O	NTNa
P099	502, 2396	500, 2390	O, N	NWi
P156	-		-	Eu-N
P141	-		-	Na-N
P221a	2419, 5818, 9190	2390, 5850, 9200	N, O	NTNa
P221b	502, 2396	500, 2390	O, N	NWi
P059	-		-	O

## Supplementary Materials

The following are available online at https://www.mdpi.com/1999-4915/12/4/478/s1. Table S1: List of plant samples included in the study, Figure S1: Diagram representing the workflow used for analysis of sequencing data, Table S2: Complete list of all PVY complete genomes used in PVY phylogenetic and recombination analyses, Table S3: Integrity of RNA extracts used for Illumina and ONT’s MinION sequencing, Table S4: Additional statistics of MinION sequencing, Figure S2: Coverage graphs obtained by mapping ONT reads to the viral reference sequence, Figure S3: Base accuracy of polyT- and random hexamers-primed ONT reads mapping to the Illumina-based final consensus sequences of the viruses detected in this study, Figure S4: Condensed maximum likelihood phylogenetic trees for different portions of PVY genomes, as identified by recombination analysis.
