# Supplementary material for: Characterization of Potato Virus Y Isolates and Assessment of Nanopore Sequencing to Detect and Genotype Potato Viruses
Source: Viruses. 2020 Apr 23;12(4):478. doi: 10.3390/v12040478 (PMC7232445; doi:10.3390/v12040478)
Supplement: Supplementary file 1 [file viruses-12-00478-s001.zip › supplementary_materials_Della Bartola_et_al_2.pdf]

Table S.1 List of plant samples included in the study. Sampling date, geographical origin and results of the molecular and serological tests are reported.

Sample ID	Sampling date	Origin	Virus detected	RT-PCR PVY genotype	ELISA PVY Pab	ELISA PVY Mab
P001	26/06/2017	Co. Carlow	PVY	NWi	+	O/C
P002	26/06/2017	Co. Carlow	-		-	-
P003	26/06/2017	Co. Carlow	PVY	NTNa	+	N
P004	26/06/2017	Co. Carlow	PVY	NTNa	+	N
P005	26/06/2017	Co. Carlow	PVY	NWi	+	O/C
P006	26/06/2017	Co. Carlow	PVY	NTNa	+	N
P007	26/06/2017	Co. Carlow	-	-	-	-
P008	26/06/2017	Co. Carlow	-	-	-	-
P009	26/06/2017	Co. Carlow	PVY	NWi	+	O/C
P010	26/06/2017	Co. Carlow	PVY	NWi	+	O/C
P011	26/06/2017	Co. Carlow	-	-	-	-
P012	26/06/2017	Co. Carlow	-	-	-	-
P013	26/06/2017	Co. Carlow	PVY	NWi	+	O/C
P014	26/06/2017	Co. Carlow	PVY	NWi	+	O/C
P015	26/06/2017	Co. Carlow	PVY	NTNa	+	N
P016	26/06/2017	Co. Carlow	-	-	-	-
P017	26/06/2017	Co. Carlow	-	-	-	-
P018	26/06/2017	Co. Carlow	PVY	NTNa	+	N
P019	26/06/2017	Co. Carlow	PVY	NTNa	+	N
P020	26/06/2017	Co. Carlow	-	-	-	-
P021	26/06/2017	Co. Carlow	PVY	NTNa	+	N
P022	26/06/2017	Co. Carlow	PVY	-	-	-
P023	26/06/2017	Co. Carlow	-	NWi	+	O/C
P024	26/06/2017	Co. Carlow	PVY	NWi	+	O/C
P025	26/06/2017	Co. Carlow	PVY	NTNa	+	N
P026	26/06/2017	Co. Carlow	PVY	O	+	O/C
P027	26/06/2017	Co. Carlow	PVY	O + NWi	+	O/C
P028	26/06/2017	Co. Carlow	-	NWi	-	O/C
P029	05/07/2017	Co. Carlow	PVY	NTNa	+	N
P030	05/07/2017	Co. Carlow	PVY	NTNa	+	N
P031	05/07/2017	Co. Carlow	PVY	Recombinant mixed infection	+	O/C + N
P032	05/07/2017	Co. Carlow	PVY	NTNa	+	N
P033	05/07/2017	Co. Carlow	PVY	Nwi	+	O/C
P034	05/07/2017	Co. Carlow	PVY	Recombinant mixed infection	+	O/C + N
P035	05/07/2017	Co. Carlow	PVY	NWi	+	O/C
P036	05/07/2017	Co. Carlow	PVY	NWi	+	O/C
P037	05/07/2017	Co. Carlow	PVY	NWi	+	O/C
P038	05/07/2017	Co. Carlow	-	-	-	-
P039	05/07/2017	Co. Carlow	PVY	NWi	+	O/C
P040	05/07/2017	Co. Carlow	PVY	NWi	+	O/C
P041	04/08/2017	Co. Carlow	-	-	-	-
P042	04/08/2017	Co. Carlow	PVY	NTNa	+	N
P043	04/08/2017	Co. Carlow	PVY	Recombinant mixed infection	+	O/C + N
P044	04/08/2017	Co. Carlow	PVY	Recombinant mixed infection	+	O/C + N
P045	04/08/2017	Co. Carlow	PVY	NTNa	+	N
P046	04/08/2017	Co. Carlow	PVY	NTNa	+	N
P047	04/08/2017	Co. Carlow	PVY	NTNa	+	N

Sample ID	Sampling date	Origin	Virus detected	RT-PCR PVY genotype	ELISA PVY Pab	ELISA PVY Mab
P048	04/08/2017	Co. Carlow	PVY	NTNa	+	N
P049	04/08/2017	Co. Carlow	PVY	NWi	+	O/C
P050	04/08/2017	Co. Carlow	PVY	NWi	+	O/C
P051	17/08/2017	Co. Carlow	PVY + PVX	Recombinant mixed infection	+	O/C + N
P052	17/08/2017	Co. Carlow	PVY + PVS + PVX	O	+	O/C
P053	17/08/2017	Co. Carlow	PVY + PVS + PVX	O	+	O/C
P054	17/08/2017	Co. Carlow	PVY + PVS + PVX	NTNA + O	+	O/C + N
P055	17/08/2017	Co. Carlow	PVY + PVS + PVX	O	+	O/C
P056	17/08/2017	Co. Carlow	PVY + PVS	NWi	+	O/C
P057	17/08/2017	Co. Carlow	PVY + PVX	NWi	+	O/C
P058	17/08/2017	Co. Carlow	PVS + PVX	-	-	-
P059	17/08/2017	Co. Carlow	PVY + PVS + PVX	O	+	O/C
P060	17/08/2017	Co. Carlow	PVY + PVS + PVX	NTNa	+	N
P061	17/08/2017	Co. Carlow	PVY + PVS + PVA	Recombinant mixed infection	+	O/C + N
P062	17/08/2017	Co. Carlow	PVY + PVS	NTNA + O	+	O/C + N
P063	17/08/2017	Co. Carlow	PVY + PVS	O + NWi	+	O/C
P064	17/08/2017	Co. Carlow	PVY	NTNa	+	N
P065	17/08/2017	Co. Carlow	PVY + PVS	NTNa	+	N
P066	17/08/2017	Co. Carlow	-	-	-	-
P067	17/08/2017	Co. Carlow	PVY + PVS + PVX	NTNa + O	+	O/C + N
P068	17/08/2017	Co. Carlow	PVY	NWi	+	O/C
P069	17/08/2017	Co. Carlow	PVY + PVS + PVX	NWi	+	O/C
P070	17/08/2017	Co. Carlow	PVY	NWi	+	O/C
P071	18/08/2017	Co. Kilkenny	PVY	NTNa	+	N
P073	18/08/2017	Co. Kilkenny	-	-	-	-
P074	18/08/2017	Co. Kilkenny	-	-	-	-
P075	18/08/2017	Co. Kilkenny	PVY	NWi	+	O/C
P076	18/08/2017	Co. Kilkenny	PVY	NTNa	+	N
P077	18/08/2017	Co. Kilkenny	PVY	NWi	+	O/C
P079	18/08/2017	Co. Kilkenny	PVY	NWi	+	O/C
P080	18/08/2017	Co. Kilkenny	PVY	NTNa	+	N
P082	18/08/2017	Co. Kilkenny	PVY	NWi	+	O/C
P083	18/08/2017	Co. Kilkenny	PVY	NWi	+	O/C
P084	18/08/2017	Co. Kilkenny	PVY	Recombinant mixed infection	+	O/C + N
P085	18/08/2017	Co. Kilkenny	PVY	Recombinant mixed infection	+	O/C + N
P086	18/08/2017	Co. Kilkenny	PVY	Recombinant mixed infection	+	O/C + N
P087	18/08/2017	Co. Kilkenny	PVY	Recombinant mixed infection	+	O/C + N
P088	18/08/2017	Co. Kilkenny	-	-	-	-
P089	18/08/2017	Co. Kilkenny	PVY	NWi	+	O/C
P090	18/08/2017	Co. Kilkenny	PVY	NTNa	+	N
P091	18/08/2017	Co. Kilkenny	PVY	NTNa	+	N
P092	18/08/2017	Co. Kilkenny	PVY	NWi	+	O/C
P093	18/08/2017	Co. Kilkenny	PVY	NTNa	+	N
P094	18/08/2017	Co. Kilkenny	-	-	-	-
P095	11/09/2017	Co. Carlow	-	-	-	-
P096	11/09/2017	Co. Carlow	-	-	-	-
P097	11/09/2017	Co. Carlow	PVY	NTNa	+	N
P098	11/09/2017	Co. Carlow	PVY	Recombinant mixed infection	+	O/C + N

Sample ID	Sampling date	Origin	Virus detected	RT-PCR PVY genotype	ELISA PVY Pab	ELISA PVY Mab
P099	11/09/2017	Co. Carlow	PVY	NWi	+	O/C
P100	11/09/2017	Co. Carlow	PVY	NTNa + O	+	O/C + N
P105	11/07/2018	Co. Louth	PVY	NTNa	+	N
P106	11/07/2018	Co. Louth	PVY	NTNa	+	N
P107	04/07/2018	Co. Louth	PVY	NTNa	+	N
P108	04/07/2018	Co. Louth	PVY	NTNa	+	N
P109	05/07/2018	Co. Louth	PVY	NTNa	+	N
P110	05/07/2018	Co. Louth	PVY	NTNa	+	N
P111	05/07/2018	Co. Louth	PVY	NTNa	+	N
P112	05/07/2018	Co. Louth	PVY	NTNa	+	N
P113	05/07/2018	Co. Louth	PVY	NTNa	+	N
P114	05/07/2018	Co. Louth	PVY	NTNa	+	N
P115	05/07/2018	Co. Louth	PVY	NTNa	+	N
P116	05/07/2018	Co. Louth	PVY	NTNa	+	N
P117	11/07/2018	Co. Cork	PVY	NTNa	+	N
P118	11/07/2018	Co. Cork	-	-	-	-
P119	11/07/2018	Co. Cork	PVY	NTNa	+	N
P120	11/07/2018	Co. Cork	PVA	-	-	-
P121	11/07/2018	Co. Cork	PVA	-	-	-
P122	11/07/2018	Co. Cork	PVY	NTNa	+	N
P123	11/07/2018	Co. Cork	PVA	-	-	-
P124	11/07/2018	Co. Cork	PVA	-	-	-
P125	11/07/2018	Co. Cork	PVY	NTNa	+	N
P126	11/07/2018	Co. Cork	PVA	-	-	-
P127	13/07/2018	Co. Louth	PVY	NTNa	+	N
P128	13/07/2018	Co. Louth	PVY	NTNa	+	N
P129	13/07/2018	Co. Louth	PVY	NTNa	+	N
P130	13/07/2018	Co. Louth	PVY	NTNa	+	N
P131	13/07/2018	Co. Louth	PVY	NTNa	+	N
P132	13/07/2018	Co. Louth	PVY	NTNa	+	N
P133	13/07/2018	Co. Louth	PVY	NTNa	+	N
P134	13/07/2018	Co. Louth	PVY	NTNa	+	N
P135	13/07/2018	Co. Louth	PVY	NTNa	+	N
P136	13/07/2018	Co. Louth	PVY	NTNa	+	N
P137	13/07/2018	Co. Louth	PVY	NTNa	+	N
P138	13/07/2018	Co. Louth	PVY	NTNa	+	N
P139	13/07/2018	Co. Louth	PVY	NTNa	+	N
P140	13/07/2018	Co. Louth	PVY	NTNa	+	N
P141	16/07/2018	Co. Donegal	PVY	NA-N	+	N
P142	16/07/2018	Co. Donegal	PVY	NTNa	+	N
P143	16/07/2018	Co. Donegal	PVY	NTNa	+	N
P144	18/07/2018	Co. Donegal	PVY	NTNa	+	N
P145	18/07/2018	Co. Donegal	PVY	NTNa	+	N
P146	18/07/2018	Co. Donegal	PVY	NTNa	+	N
P147	18/07/2018	Co. Donegal	PVY	NTNa	+	N
P148	18/07/2018	Co. Donegal	PVY	NTNa	+	N
P149	18/07/2018	Co. Donegal	PVY	NTNa	+	N
P150	17/07/2018	Co. Louth	PVY	NTNa	+	N
P151	17/07/2018	Co. Louth	PVY	NTNa	+	N
P152	17/07/2018	Co. Louth	-	-	-	-
P153	17/07/2018	Co. Louth	PVY	NTNa	+	N
P154	17/07/2018	Co. Louth	PVY	NTNa	+	N

Sample ID	Sampling date	Origin	Virus detected	RT-PCR PVY genotype	ELISA PVY Pab	ELISA PVY Mab
P155	25/07/2018	Co. Offaly	PVY	NA-N	+	N
P156	25/07/2018	Co. Offaly	PVY	N	+	N
P157	25/07/2018	Co. Offaly	PVY	NTNa	+	N
P158	25/07/2018	Co. Offaly	PVY	NA-N	+	N
P159	25/07/2018	Co. Offaly	PVY	NTNa	+	N
P160	25/07/2018	Co. Offaly	-	-	-	-
P161	25/07/2018	Co. Offaly	PVY	NTNa	+	N
P162	25/07/2018	Co. Offaly	PVY	NTNa	+	N
P163	30/07/2018	Co. Offaly	-	-	-	-
P164	30/07/2018	Co. Offaly	-	-	-	-
P165	30/07/2018	Co. Offaly	-	-	-	-
P166	30/07/2018	Co. Offaly	PLRV	-	-	-
P167	30/07/2018	Co. Offaly	-	-	-	-
P168	30/07/2018	Co. Offaly	-	-	-	-
P169	30/07/2018	Co. Offaly	-	-	-	-
P170	30/07/2018	Co. Offaly	-	-	-	-
P187	31/07/2018	Co. Wexford	PVY + PVA	NTNa	+	N
P188	31/07/2018	Co. Wexford	PVY + PVA	NTNa	+	N
P189	31/07/2018	Co. Wexford	PVY + PVA	NTNa	+	N
P190	31/07/2018	Co. Wexford	PVY + PVA	NTNa + O	+	O/C + N
P191	31/07/2018	Co. Wexford	PVY + PVA	NTNa + O	+	O/C + N
P192	31/07/2018	Co. Wexford	PVY	NTNa	+	N
P193	31/07/2018	Co. Wexford	PVY	NTNa	+	N
P194	31/07/2018	Co. Wexford	PVY	NTNa	+	N
P195	27/07/2018	Co. Louth	PVY	NTNa	+	N
P196	27/07/2018	Co. Louth	PVY	NTNa	+	N
P197	27/07/2018	Co. Louth	PVY	NTNa	+	N
P198	27/07/2018	Co. Louth	PVY	NTNa	+	N
P199	02/08/2018	Co. Offaly	PVY	NTNa	+	N
P200	02/08/2018	Co. Offaly	PVY	NA-N	+	N
P201	02/08/2018	Co. Offaly	PVY	NTNa	+	N
P202	02/08/2018	Co. Offaly	PVY	NA-N	+	N
P203	02/08/2018	Co. Offaly	PVY	NTNa	+	N
P204	02/08/2018	Co. Offaly	PVY	NTNa	+	N
P205	02/08/2018	Co. Offaly	PVY	NTNa	+	N
P206	02/08/2018	Co. Offaly	PVY	NTNa	+	N
P207	02/08/2018	Co. Offaly	PVY	NTNa	+	N
P208	02/08/2018	Co. Offaly	PVY	NTNa	+	N
P209	02/08/2018	Co. Offaly	PVY	NTNa	+	N
P210	02/08/2018	Co. Offaly	PVY	NTNa	+	N
P211	08/08/2018	Co. Carlow	PVY	O + NA-N	+	O/C + N
P212	08/08/2018	Co. Carlow	PVY	O + Nwi	+	O/C
P213	08/08/2018	Co. Carlow	PVY	Nwi	+	O/C
P214	08/08/2018	Co. Carlow	PVY	O + Na-N	+	O/C + N
P215	12/09/2018	Co. Carlow	-	-	-	-
P217	12/09/2018	Co. Carlow	PVY + PVS	Nwi	+	O/C
P218	12/09/2018	Co. Carlow	-	-	-	-
P219	12/09/2018	Co. Carlow	PVY + PVS	Nwi	+	O/C
P220	12/09/2018	Co. Carlow	PVY + PVS	O	+	O/C
P221	12/09/2018	Co. Carlow	PVY	Recombinant mixed infection	+	O/C + N
P222	12/09/2018	Co. Carlow	PVY	NTNa	+	N

Sample ID	Sampling date	Origin	Virus detected	RT-PCR PVY genotype	ELISA PVY Pab	ELISA PVY Mab
P223	17/09/2018	Co. Carlow	PVY	NTNa	+	N
P224	17/09/2018	Co. Carlow	PVY	Recombinant mixed infection	+	O/C + N

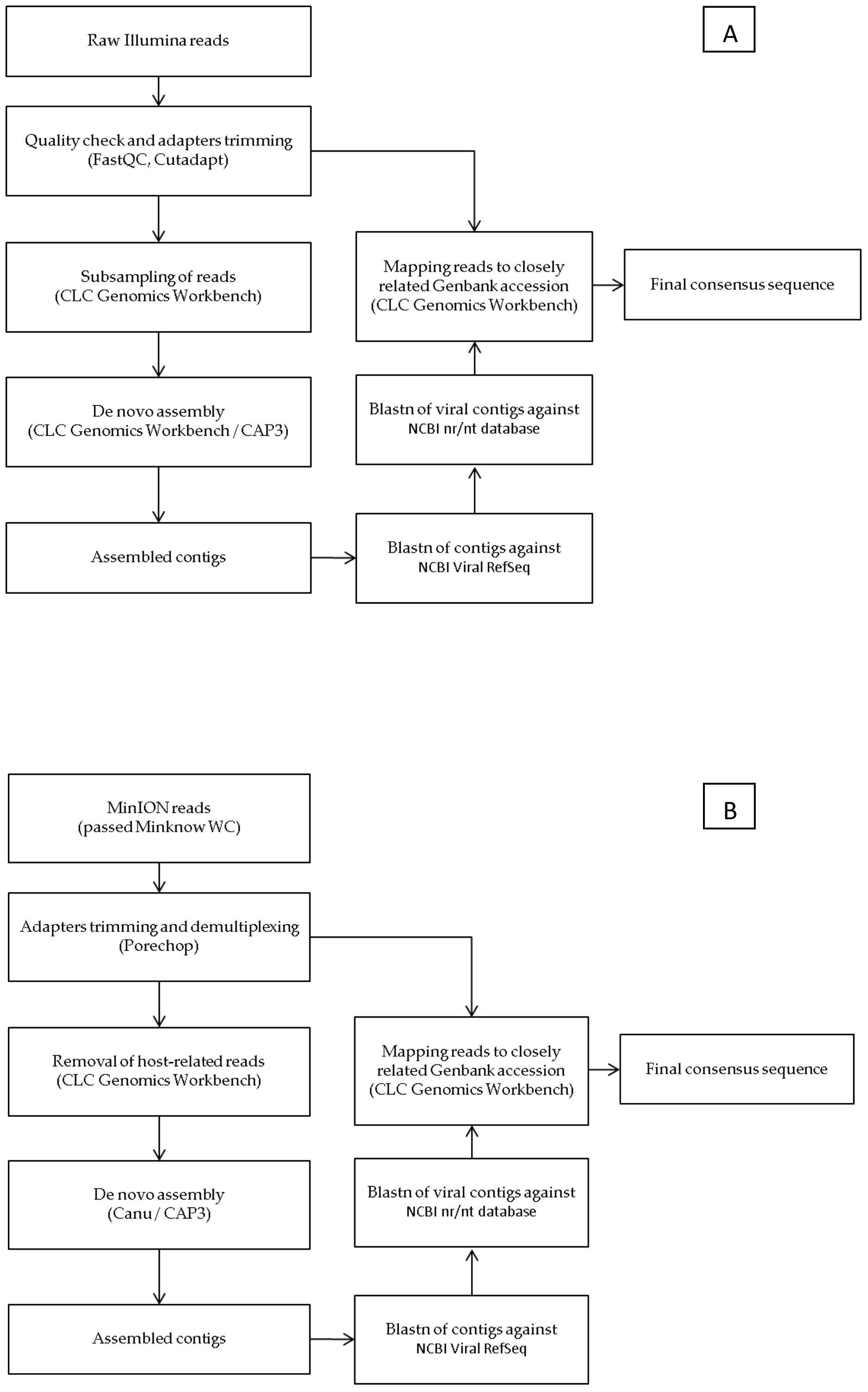

Figure S.1 Diagram representing the workflow used for analysis of sequencing data (A. Illumina reads; B. MinION reads)

Table S.2. Complete list of all PVY complete genomes used in PVY phylogenetic and recombination analyses.

#	Isolate Name	GenBank Accession	PVY Strain [14]	Origin
1	PVYOUK	JX424837	O	Finland
2	IUNG_15	JF927763	NTNa	Poland
3	IUNG_5	JF927753	NWi	Poland
4	N_605	X97895	Eu-N	Switzerland
8	SASA-61	AJ585198	Na-N	UK
9	NTNON92	AB331519	Na-N	Japan
10	SON41	AJ439544	C	France
11	MN	AF463399	C	USA
12	SCRI_O	AJ585196	O	UK
13	CO284	HQ912905	O5	USA
14	DEL3	KP691325	O5	USA
15	Mont	AY884983	Eu-N	USA
16	SASA-207	AJ584851	N:O (long)	UK
17	34_01	AJ890342	NTNb	Poland
18	SYR_NB_16	AB270705	SYR I	Syria
19	SYR_II_2_8	AB461451	SYR II	Syria
20	HC_2quan	HM590406	NTNb	China
21	SYR_III_L4	AB461454	SYR III	Syria
22	IUNG_14	JF927762	N:O short	Poland
23	ME162_CN	JQ971975	NE 11 long	USA
24	Eu_12Jp	AB702945	NTNa	Japan
25	VNP415	HG810951	SYR II	Vietnam
26	ShX14	KJ634024	SYR I	China
27	ID26	KY847955	NE 11 short	USA
28	ID26	KY847996	NWi	USA
29	Egypt7	KY863549	SYR III	Egypt

Table S.3 Integrity of RNA extracts used for Illumina and ONT's MinION sequencing.

Plant Sample	RIN Value[1]	rRNA ratio[1]
P026	3.4	0.1
P097	5.0	0.6
P099	5.7	1.0
P156	2.1	0.1
P141	2.1	0.1
P221	3.5	0.4
P166	2.3	0.1
P059	4.8	1.2

[1] as assessed using Agilent RNA 6000 Pico chip on a 2100 Bioanalyzer (Agilent Technologies)

Table S.4 Additional statistics of MinION sequencing. Number of raw reads per sequencing run, number of reads per sample after trimming/demultiplexing and after removal of host-related reads. The percentage of reads mapping to the viruse(s) detected is also reported, together with the maximum and average length of virus-related reads.

Sequencing run (No. of raw reads[1])	Sample	Library	No. of reads[2]	No. reads after hostfiltering - %	% of reads mapped to virus reference (accession no.)	Maximum / average length of mapped reads
PolyT Run 1 (9463852)	P026	polyT	1445021	29296 – 2.03%	0.71% (JX424937)	6685 / 1007.36 nt
	P097	polyT	1567476	51355 – 3.28%	0.54% (JF927763)	3801 / 572.63 nt
	P099	polyT	1676243	65574 – 3.91%	0.98% (JF927753)	4454 / 586.15 nt
	P156	polyT	1346123	76483 – 5.68%	2.84% (X97895)	6250 / 494.73 nt
PolyT Run 2 (7963207)	P141	polyT	936278	48821 – 5.21%	2.74% (AB331517)	5476 / 568.69 nt
	P221	polyT	1005366	58253 – 5.79%	1.08% (JF927763)<br>1.53% (JF927754)	4491 / 458.64 nt<br>5697 / 502.09 nt
	P166	polyT	668443	26926 – 4.03%	0.24% (AY138970)	2442 / 318.54 nt
	P059	polyT	865252	142641 – 16.49%	1.63% (MF418030)<br>11.51% (KR605396)<br>0.23% (MH795851)	3885 / 528.01 nt<br>4397 / 462.60 nt<br>2290 / 423.91 nt
RH Run 1 (9286022)	P026	RH	1679262	13637 – 0.81%	0.33%(JX424937)	5483 / 947.73 nt
	P097	RH	1707512	19707 – 1.15%	0.25% (JF927763)	4211 / 613.31 nt
	P099	RH	1934615	30732 – 1.57%	0.53% (JF927753)	5007 / 587.88 nt
	P156	RH	1109307	31345 – 2.83%	1.58% (X97895)	4382 / 536.23 nt
RH Run 2 (9062001)	P141	RH	1290010	29782 – 2.31%	1.43% (AB331517)	5601 / 640.82 nt
	P221	RH	1239910	29734 – 2.40%	0.57% (JF927763)<br>0.76% (JF927754)	6467 / 559.37 nt<br>5724 / 581.00 nt
	P166	RH	1146691	15110 – 1.32%	0.24% (AY138970)	2418 / 338.90 nt
	P059	RH	1140049	107460 – 9.43%	1.79% (MF418030)<br>6.50% (KR605396)<br>0.13% (MH795851)	4784 / 612.05 nt<br>5681 / 584.20 nt<br>2652 / 493.29 nt

[1] MinION reads that passed the Minknow quality filtering

[2] after Porechop trimming and demultiplexing

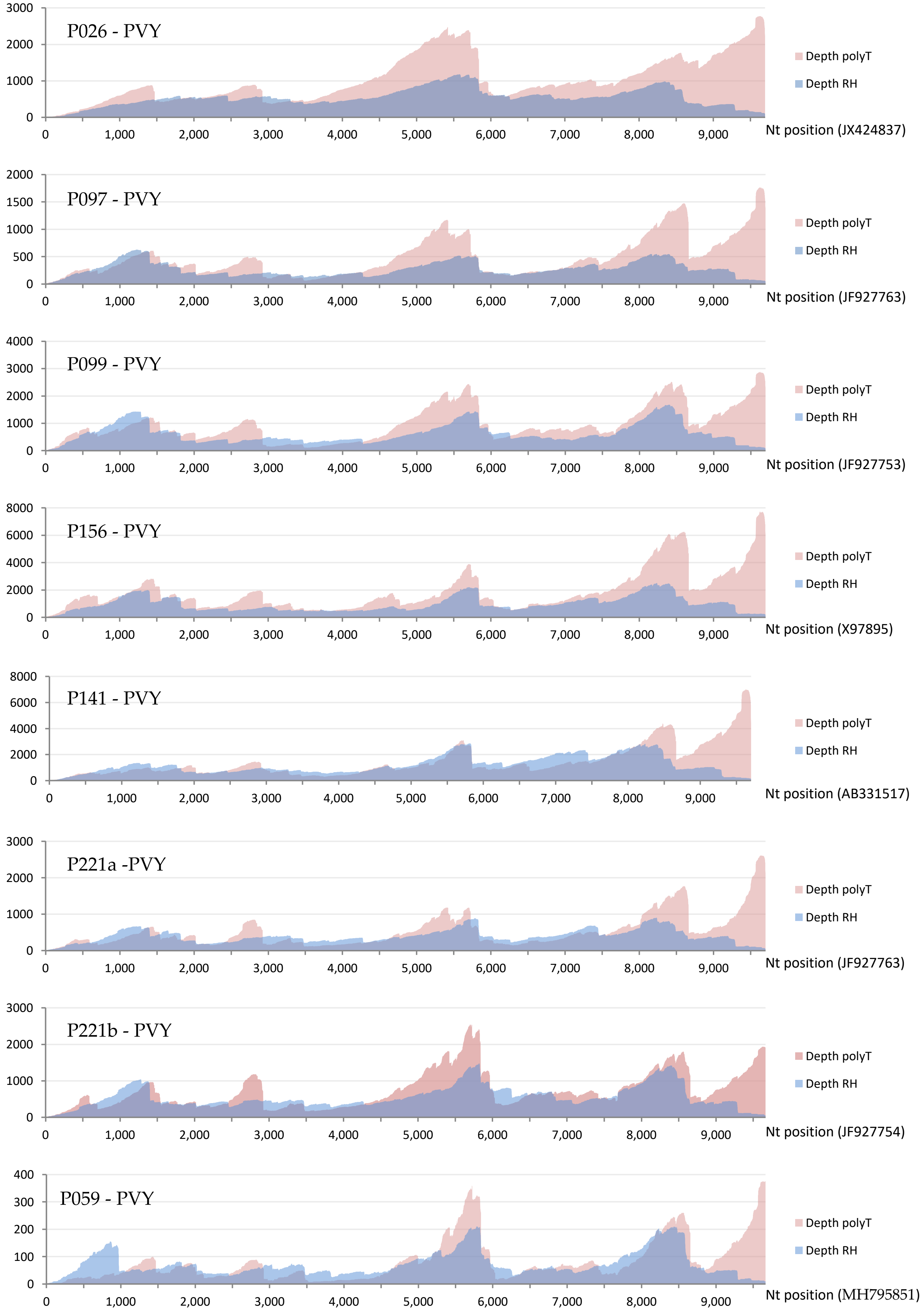

P026 - PVY
Depth polyT
Depth RH
Nt position (JX424837)
P097 - PVY
Depth polyT
Depth RH
Nt position (JF927763)
P099 - PVY
Depth polyT
Depth RH
Nt position (JF927753)
P156 - PVY
Depth polyT
Depth RH
Nt position (X97895)
P141 - PVY
Depth polyT
Depth RH
Nt position (AB331517)
P221a -PVY
Depth polyT
Depth RH
Nt position (JF927763)
P221b - PVY
Depth polyT
Depth RH
Nt position (JF927754)
P059 - PVY
Depth polyT
Depth RH
Nt position (MH795851)

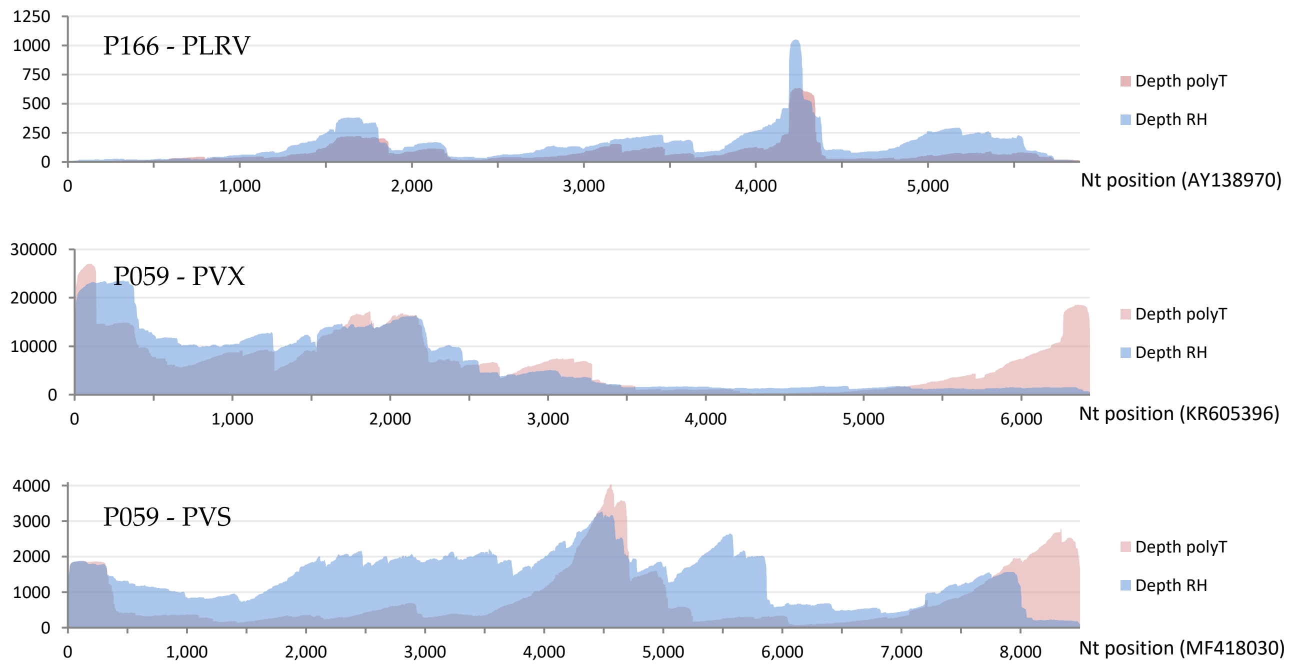

Figure S.2. (continued from previous page) Coverage graphs obtained by mapping ONT reads to the viral reference sequence (GenBank accession number in parenthesis on the right side of every graph). Per each sample, sequencing depths (y axis) at every nt position (x axis) from polyT- (pink) and random hexamers-primed (blue) libraries are plotted in a single graph. For clarity of display, the y axis on each graph scaled accordingly.

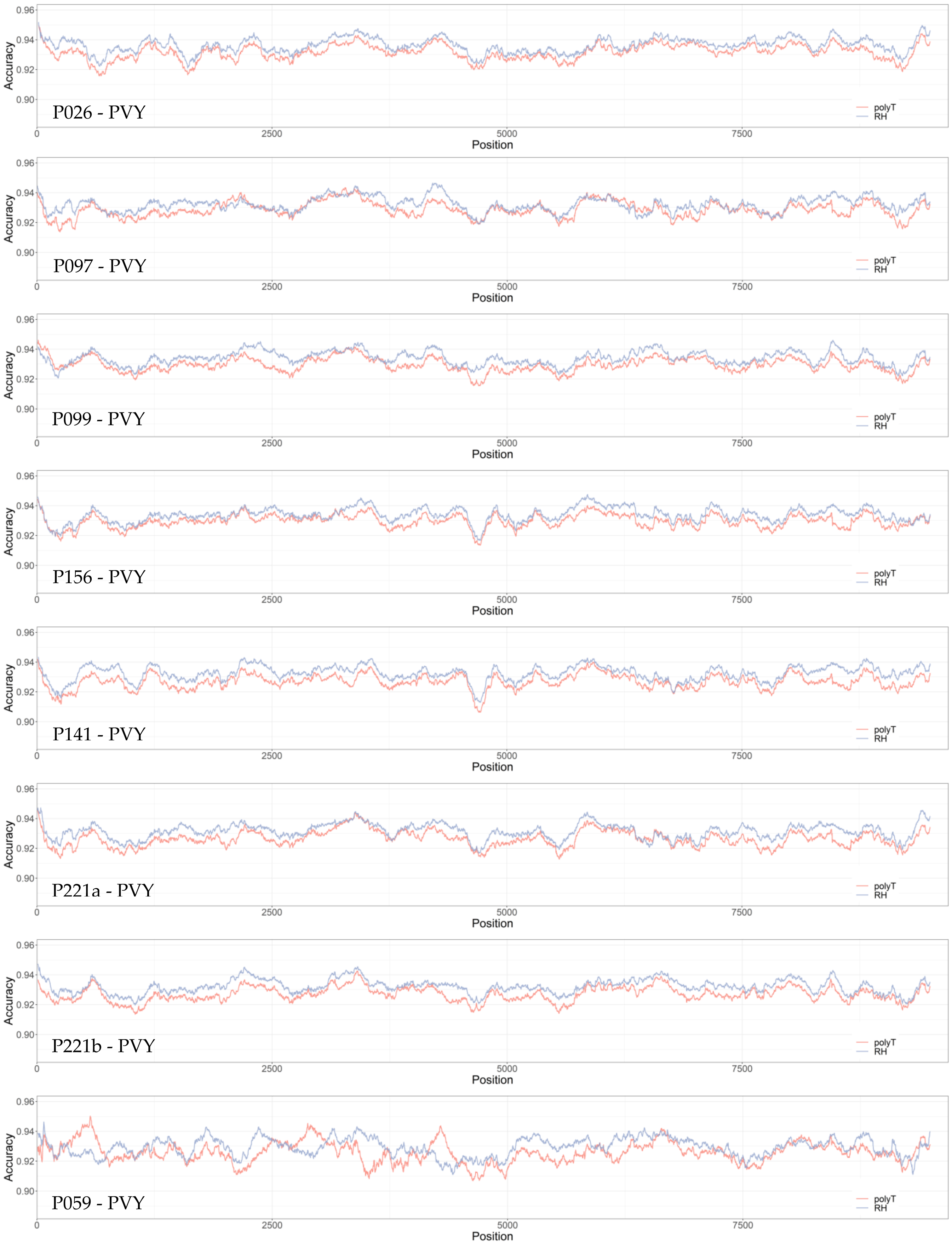
P026 - PVY
P097 - PVY
P099 - PVY
P156 - PVY
P141 - PVY
P221a - PVY
P221b - PVY
P059 - PVY
Accuracy
0.96
0.94
0.92
0.90
Position
0
2500
5000
7500
polyT
RH

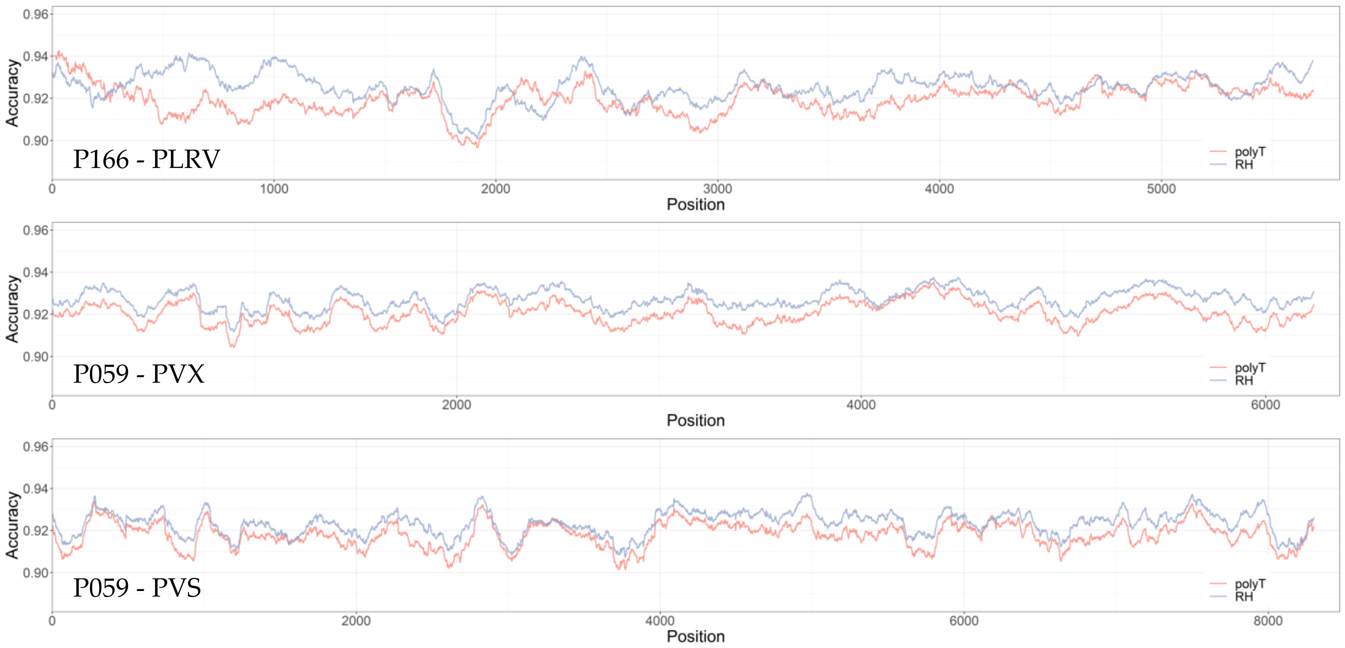

Figure S.3. (continued from previous page) Base accuracy of polyT- and random hexamers-primed ONT reads mapping to the Illumina-based final consensus sequences of the viruses detected in this study. Accuracy is expressed as percentage of matches over coverage at each nt position.

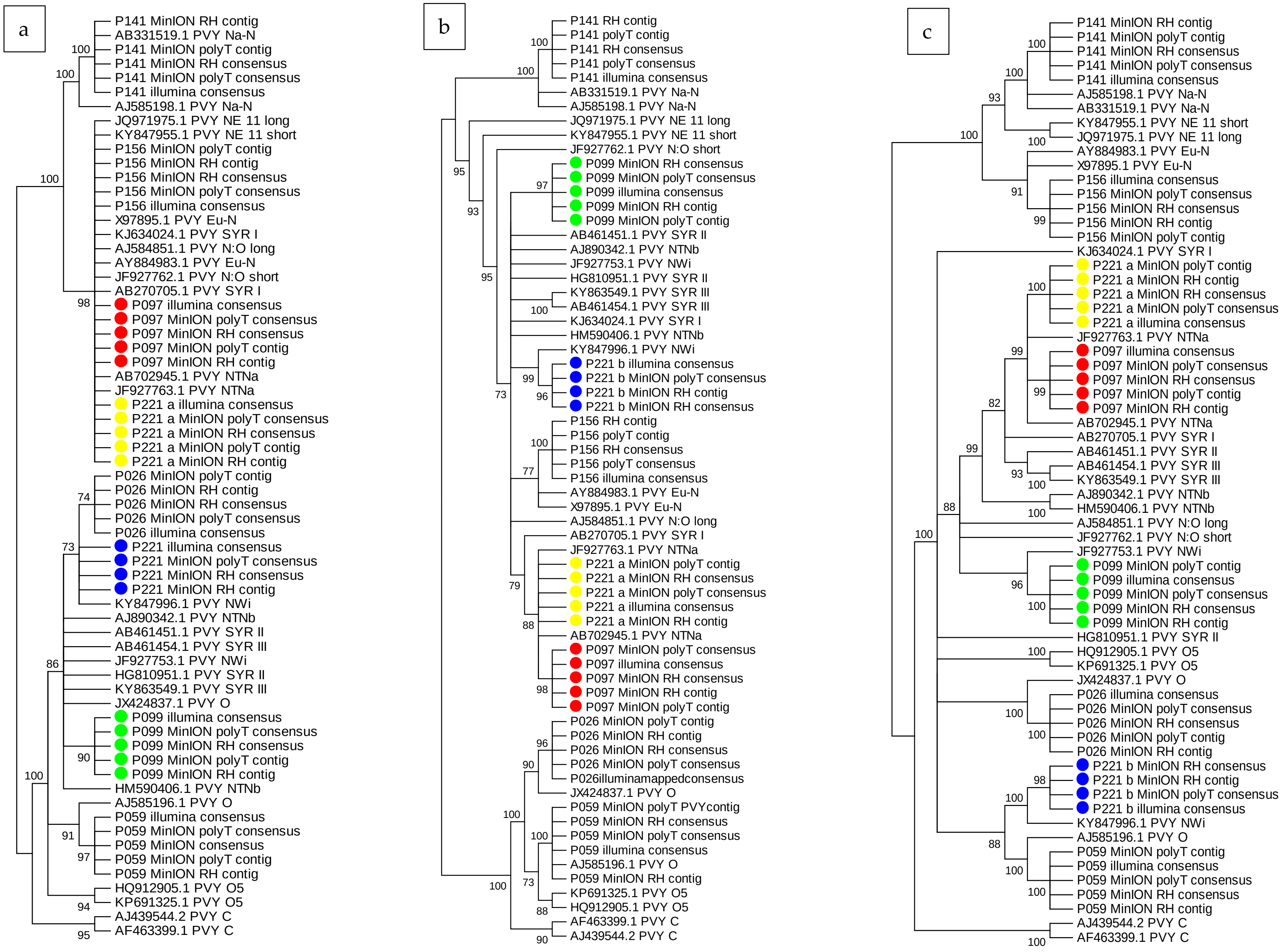
a
P141 MinION RH contig
AB331519.1 PVY Na-N
P141 MinION polyT contig
P141 MinION RH consensus
P141 MinION polyT consensus
P141 illumina consensus
AJ585198.1 PVY Na-N
JQ971975.1 PVY NE 11 long
KY847955.1 PVY NE 11 short
P156 MinION polyT contig
P156 MinION RH contig
P156 MinION RH consensus
P156 MinION polyT consensus
P156 illumina consensus
X97895.1 PVY Eu-N
KJ634024.1 PVY SYR I
AJ584851.1 PVY N:O long
AY884983.1 PVY Eu-N
JF927762.1 PVY N:O short
AB270705.1 PVY SYR I
P097 illumina consensus
P097 MinION polyT consensus
P097 MinION RH consensus
P097 MinION polyT contig
P097 MinION RH contig
AB702945.1 PVY NTNa
JF927763.1 PVY NTNa
P221 a illumina consensus
P221 a MinION polyT consensus
P221 a MinION RH consensus
P221 a MinION polyT contig
P221 a MinION RH contig
P026 MinION polyT contig
P026 MinION RH contig
P026 MinION RH consensus
P026 MinION polyT consensus
P026 illumina consensus
P221 illumina consensus
P221 MinION polyT consensus
P221 MinION RH consensus
P221 MinION RH contig
KY847996.1 PVY NWi
AJ890342.1 PVY NTNb
AB461451.1 PVY SYR II
AB461454.1 PVY SYR III
JF927753.1 PVY NWi
HG810951.1 PVY SYR II
KY863549.1 PVY SYR III
JX424837.1 PVY O
P099 illumina consensus
P099 MinION polyT consensus
P099 MinION RH consensus
P099 MinION polyT contig
P099 MinION RH contig
HM590406.1 PVY NTNb
AJ585196.1 PVY O
P059 illumina consensus
P059 MinION polyT consensus
P059 MinION consensus
P059 MinION polyT contig
P059 MinION RH contig
HQ912905.1 PVY O5
KP691325.1 PVY O5
AJ439544.2 PVY C
AF463399.1 PVY C
b
P141 RH contig
P141 polyT contig
P141 RH consensus
P141 polyT consensus
P141 illumina consensus
AB331519.1 PVY Na-N
AJ585198.1 PVY Na-N
JQ971975.1 PVY NE 11 long
KY847955.1 PVY NE 11 short
JF927762.1 PVY N:O short
P099 MinION RH consensus
P099 MinION polyT consensus
P099 illumina consensus
P099 MinION RH contig
P099 MinION polyT contig
AB461451.1 PVY SYR II
AJ890342.1 PVY NTNb
JF927753.1 PVY NWi
HG810951.1 PVY SYR II
KY863549.1 PVY SYR III
AB461454.1 PVY SYR III
KJ634024.1 PVY SYR I
HM590406.1 PVY NTNb
KY847996.1 PVY NWi
P221 b illumina consensus
P221 b MinION polyT consensus
P221 b MinION RH contig
P221 b MinION RH consensus
P156 RH contig
P156 polyT contig
P156 RH consensus
P156 polyT consensus
P156 illumina consensus
AY884983.1 PVY Eu-N
X97895.1 PVY Eu-N
AJ584851.1 PVY N:O long
AB270705.1 PVY SYR I
JF927763.1 PVY NTNa
P221 a MinION polyT contig
P221 a MinION RH consensus
P221 a MinION polyT consensus
P221 a illumina consensus
P221 a MinION RH contig
AB702945.1 PVY NTNa
P097 MinION polyT consensus
P097 illumina consensus
P097 MinION RH consensus
P097 MinION RH contig
P097 MinION polyT contig
P026 MinION polyT contig
P026 MinION RH contig
P026 MinION RH consensus
P026 MinION polyT consensus
P026illuminamappedconsensus
JX424837.1 PVY O
P059 MinION polyT PVYcontig
P059 MinION RH consensus
P059 MinION polyT consensus
P059 illumina consensus
AJ585196.1 PVY O
P059 MinION RH contig
KP691325.1 PVY O5
HQ912905.1 PVY O5
AF463399.1 PVY C
AJ439544.2 PVY C
c
P141 MinION RH contig
P141 MinION polyT contig
P141 MinION RH consensus
P141 MinION polyT consensus
P141 illumina consensus
AJ585198.1 PVY Na-N
AB331519.1 PVY Na-N
KY847955.1 PVY NE 11 short
JQ971975.1 PVY NE 11 long
AY884983.1 PVY Eu-N
X97895.1 PVY Eu-N
P156 illumina consensus
P156 MinION polyT consensus
P156 MinION RH consensus
P156 MinION RH contig
P156 MinION polyT contig
KJ634024.1 PVY SYR I
P221 a MinION polyT contig
P221 a MinION RH contig
P221 a MinION RH consensus
P221 a MinION polyT consensus
P221 a illumina consensus
JF927763.1 PVY NTNa
P097 illumina consensus
P097 MinION polyT consensus
P097 MinION RH consensus
P097 MinION polyT contig
P097 MinION RH contig
AB702945.1 PVY NTNa
AB270705.1 PVY SYR I
AB461451.1 PVY SYR II
AB461454.1 PVY SYR III
KY863549.1 PVY SYR III
AJ890342.1 PVY NTNb
HM590406.1 PVY NTNb
AJ584851.1 PVY N:O long
JF927762.1 PVY N:O short
JF927753.1 PVY NWi
P099 MinION polyT contig
P099 illumina consensus
P099 MinION polyT consensus
P099 MinION RH consensus
P099 MinION RH contig
HG810951.1 PVY SYR II
HQ912905.1 PVY O5
KP691325.1 PVY O5
JX424837.1 PVY O
P026 illumina consensus
P026 MinION polyT consensus
P026 MinION RH consensus
P026 MinION polyT contig
P026 MinION RH contig
P221 b MinION RH consensus
P221 b MinION RH contig
P221 b MinION polyT consensus
P221 b illumina consensus
KY847996.1 PVY NWi
AJ585196.1 PVY O
P059 MinION polyT contig
P059 illumina consensus
P059 MinION polyT consensus
P059 MinION RH consensus
P059 MinION RH contig
AJ439544.2 PVY C
AF463399.1 PVY C

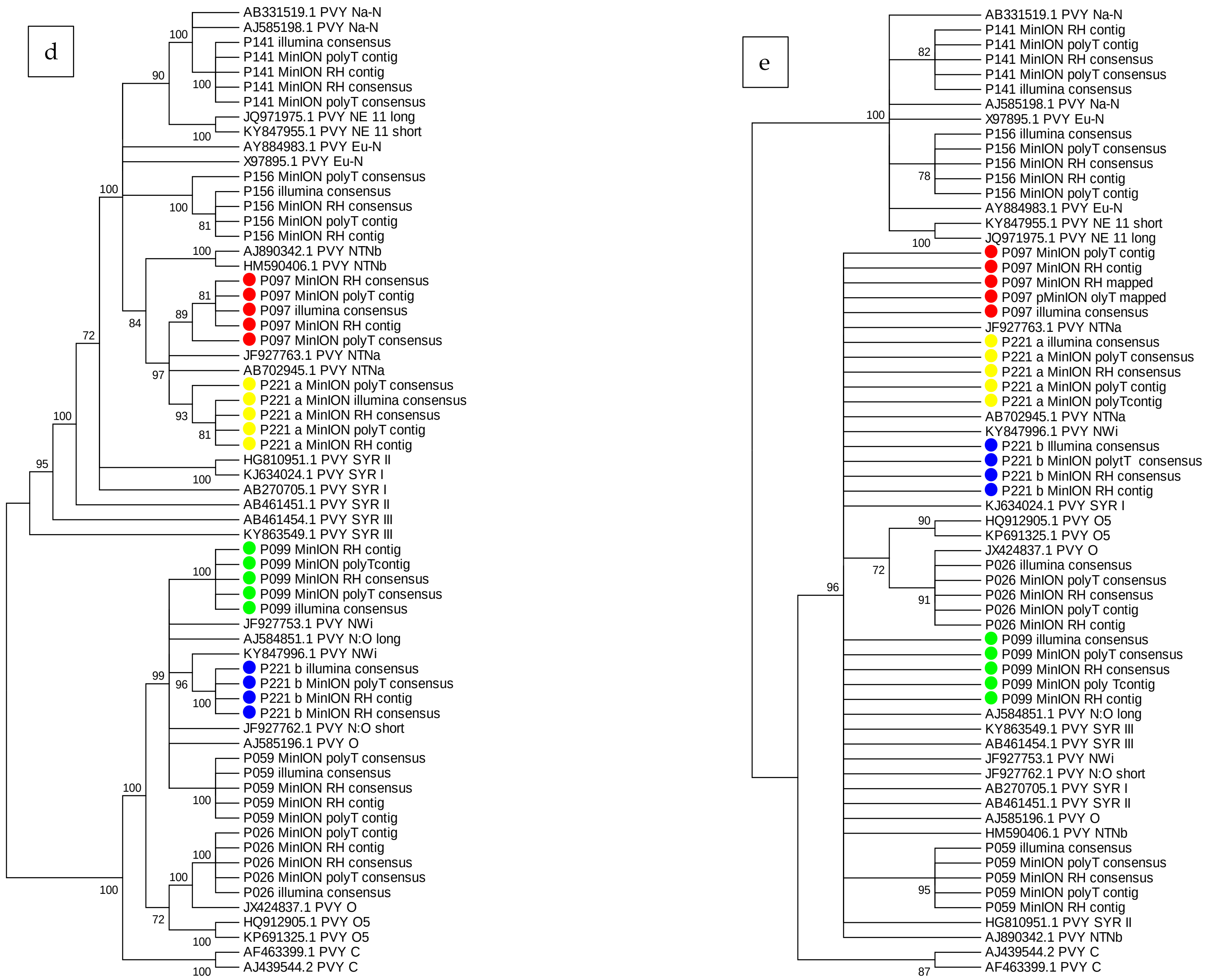

Figure S.4 (continued from previous page) Condensed maximum likelihood phylogenetic trees for different portions of PVY genomes, as identified by recombination analysis. (**a**) Section 1 from the 5′ end to nucleotide position 500; (**b**) Section 2, nucleotide positions 501 – 2390; (**c**) Section 3, nucleotide positions 2391 – 5850; (**d**) Section 4, nucleotide positions 5851 – 9200; (**e**) Section 5, from nucleotide position 9201 the 3′ end of PVY genome. Recombinant PVY sequences obtained in this study are marked with colored dots (P097 red; P099 green; P221 a yellow; P221 b blue) Only bootstrap values higher than 70% are shown.
